# Do antibody CDR loops change conformation upon binding?

**DOI:** 10.1080/19420862.2024.2322533

**Published:** 2024-03-13

**Authors:** Chu’nan Liu, Lilian M. Denzler, Oliver E.C. Hood, Andrew C.R. Martin

**Affiliations:** Structural and Molecular Biology, Division of Biosciences, University College London, London, UK

**Keywords:** antibodies, antibody structure, complementarity determining regions, CDRs, CDR flexibility, antibody binding

## Abstract

Antibodies have increasingly been developed as drugs with over 100 now licensed in the US or EU. During development, it is often necessary to increase or reduce the affinity of an antibody and rational attempts to do so rely on having a structure of the antibody-antigen complex often obtained by modeling. The antigen-binding site consists primarily of six loops known as complementarity-determining regions (CDRs), and an open question has been whether these loops change their conformation when they bind to an antigen. Existing surveys of antibody-antigen complex structures have only examined CDR conformational change in case studies or small-scale surveys. With an increasing number of antibodies where both free and complexed structures have been deposited in the Protein Data Bank, a large-scale survey of CDR conformational change during binding is now possible. To this end, we built a dataset, AbAgDb, that currently includes 177 antibodies with high-quality CDRs, each of which has at least one bound and one unbound structure. We analyzed the conformational change of the C*α* backbone of each CDR upon binding and found that, in most cases, the CDRs (other than CDR-H3) show minimal movement, while 70.6% and 87% of CDR-H3s showed global C*α* RMSD ≤ 1.0Å and ≤ 2.0Å, respectively. We also compared bound CDR conformations with the conformational space of unbound CDRs and found most of the bound conformations are included in the unbound conformational space. In future, our results will contribute to developing insights into antibodies and new methods for modeling and docking.

## Introduction

Antibodies are increasingly used as drugs owing to their high affinity and specificity and their ability to bind targets that are undruggable with small molecule drugs. At the time of writing, there are 136 antibody-based drugs approved in the United States or European Union with 17 novel antibody therapeutics having been approved since January 2023 and 18 currently in review (Antibody Society, *Antibody therapeutics approved or in regulatory review in the EU or US*, https://www.antibodysociety.org/resources/approved-antibodies/, January 24, 2024). Antibody-based drug development relies largely on time- and cost-intensive experimental approaches, which can potentially benefit substantially from computational methods such as structure- and machine learning-based design.^1–3^ An important step in structure-based design is to identify antibody-antigen interacting sites and obtain the structure of the complex.^2^ This would allow for further engineering of the binding sites to obtain antibodies with desirable binding affinities (increased or decreased), an increase in affinity through rational design based on a modeled antibody having been first achieved by Roberts *et al*. in 1989.^4^

Antigen-binding sites are the regions of the antibody surface that bind to their cognate antigens. They consist, primarily, of six complementarity-determining regions (CDRs), or ‘hypervariable loops’, three from the heavy chain and three from the light chain.^5^ Previous surveys of CDR loop structures showed that, with the exception of CDR-H3, the mainchain conformation of the other five loops can be grouped into ‘canonical structures’ which can be identified by sequence templates.^6–8^ However, the question of whether the canonical structures, or the conformation of CDR-H3, are retained upon binding, has not been considered explicitly, and the complexed/uncomplexed state has generally been ignored in existing studies.

There are three models describing the ways in which protein–protein (including antibody-antigen) interactions can occur. First, the ‘lock-and-key’ model states that there is little conformational change upon binding. Second, the ‘induced-fit’ model suggests that the bound conformation at the interface (of one or both partners) is induced by binding, with the interface of the unbound structure(s) having a distinct and different conformation from the unbound form.^9,10^ This will incur an enthalpic penalty, as the conformation of one (or both) structures will have to move away from the energy minimum seen in the unbound conformation. Thus, some of the energy gained from binding is ‘wasted’ in stressing the conformation of one or both proteins. The third model, ‘Conformational-selection’,^11^ suggests that one, or both, structures are mobile and that structural studies have ‘frozen out’ a single conformation of the free antibody that happens to be different from that present in the complex. However, this will incur an entropic penalty unless both proteins are able to move in concert in the complex. Recent surveys of general protein–protein interactions have suggested combinations of models, including conformational-selection and induced-fit.^9^

In the case of antibodies, which undergo a rapid evolutionary process to optimize binding through somatic hypermutation, it would be reasonable to expect that germline antibodies (which need to bind a range of antigens without a need for high affinity), may fit the induced-fit or conformational-selection models, with affinity maturation leading to higher affinity through a lock-and-key interaction. Indeed, this has been supported by observations of multiple pre-existing conformations of the same antibody primarily in germline antibodies,^12^ but less frequently in mature antibodies.^13^

To aid in developing new computational methods for antibody–antigen complex prediction and for understanding antibody–antigen interactions, we built a database, AbAgDb (built upon AbDb^14^), that includes both unbound and bound conformers for each antibody. The current version contains 177 groups of antibody structures with those in the same group having the same sequence and at least one unbound and one bound conformation. We then analyzed conformational change between unbound and bound conformer pairs for each CDR. We also analyzed their binding mode by comparing bound conformations against the unbound CDR conformational space, represented by canonical structures. CDR canonical structure clusters were derived by employing a similar approach to previous studies,^7,8^ but using 1,091 CDRs from only quality-filtered unbound antibodies obtained from AbDb.

## Materials and methods

Because there may be multiple structures of the same antibody (both free and with the same or different antigens), we define the term ‘antibody’ to mean an antibody with a distinct sequence present in any such set, while we define the term ‘entry’ to refer to each individual structure present in AbDb for each antibody.

### Antibodies with both unbound and bound conformations

All files used in this work were collected from the latest release of AbDb^14^ in which file names are formatted as the four-character Protein Data Bank (PDB) code, an underscore, an integer index (to distinguish antibody entries, i.e., multiple structures within a PDB file), followed by optional characters indicating the antigen type: protein and peptide (**P**), hapten (**H**), nucleic acid (**N**). An empty antigen type character indicates an unbound entry. To non-redundantize antibodies in AbDb, sequences of all antibody structures (12,205 entries) are collected, split by chain, and merged into a single FASTA file containing 21,536 chains, used as input to CD-HIT^15^ and clustered at a sequence identity of 100%. This way, each heavy or light chain is assigned to a cluster and each conventional antibody (V_H_ + V_L_) can be represented by a pair of cluster IDs (single-chain antibodies are represented by a single cluster ID). Antibodies with the same cluster-ID (single-chain antibodies) or ID pair (normal antibodies) were grouped together as they have the same sequence. This led to 3,320 unique V_H_/V_L_ antibodies (9,622 entries) and 836 unique single-chain antibodies (2,292 entries). These were then filtered to remove any problematic antibodies that could not be numbered automatically and only those having both bound and unbound structures were retained, leading to 3,040 entries representing 559 antibodies. The non-redundantization data showing groups of identical antibody entries containing free and bound examples are provided in Supplementary File *Supp01_unbound_and_bound_abs.xlsx*.

### CDR structure quality filtering

**Figure 1.**
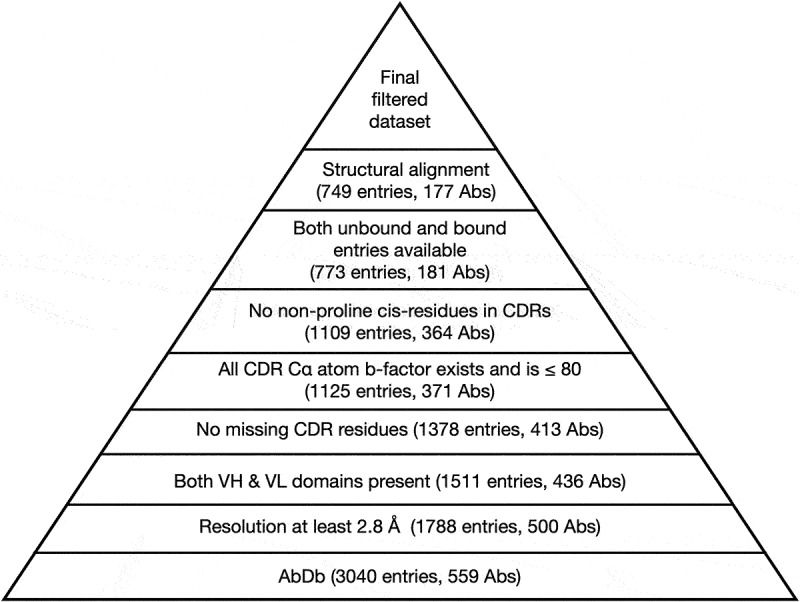
Filtering AbDb files. Starting from the bottom, we eliminate structures with resolution worse than 2.8Å and retain antibodies (Abs) that have both heavy and light variable domains, then eliminate files with missing residues in any of the six CDRs, where the C*α* atom B-factor is missing (i.e. 0) or *>* 80, or a cis non-proline residue is present in an unbound antibody, leading to 364 Abs with 1109 entries. We then retained antibodies with both unbound and bound structures (181 Abs with 773 entries) and performed global and local fitting. Finally, we eliminated unbound/bound structure pairs whose framework region showed ≥1.0Å global C*α* RMSD to minimize the impact of the framework region on CDR conformational change and followed by rechecking that both unbound and bound structures are available for an antibody, which led to the elimination of four antibodies. This led to a final set of 749 entries representing 177 antibodies. See supplementary file *Supp01_unbound_and_bound_abs.xlsx* for the initial dataset of entries with both bound and unbound structures from AbDb. See supplementary file *Supp04_antibody_filtering.xlsx* for information on entries retained and rejected at each step.

Quality filtering started with the 3,040 entries collected in the last step, numbered using the Martin scheme (a refinement of Chothia numbering in which the position of framework insertions and deletions is also structurally correct^16^). We adapted the filtering procedure from North *et al*.^8^ to create the pipeline shown in Figure 1.

The pipeline retains only entries that represent Fv structures (with both V_H_ and V_L_ domains) having a resolution of at least 2.8Å and which are of high quality; entries with missing residues, large B-factors and non-proline residues having a cis-peptide bond in any of the CDRs are eliminated. Detailed information on the final dataset is provided in Supplementary File *Supp02_primary_set.xlsx*. No NMR structures were included in the AbAgDb dataset. There were only 18 NMR structures in AbDb and only five of those contained a complete Fv (V_H_ and V_L_). Of those five, none is available as both a bound and an unbound structure. See Supplementary File *Supp03_nmr_antibody_structures.xlsx*

### CDR loop conformation analysis upon binding

We consider two types of CDR conformational change upon binding. First, the conformations of the CDRs themselves may change on binding and this can be evaluated by calculating a ‘local’ C*α* root mean square deviation (RMSD) by comparing the CDR loops in the bound and unbound structures. Alternatively, a CDR may move with respect to the supporting framework, which we refer to as loop ‘flapping’. This effect was previously observed by Bajorath *et al*.^17^ in a set of just two bound and five unbound structures. Loop flapping can be evaluated by calculating a ‘global’ RMSD where the supporting framework is fitted and the C*α* RMSD is calculated over the CDR and comparing this with the local C*α* RMSD. While the global RMSD will be affected by *both* any local conformational change and by loop flapping, a high *global* C*α* RMSD with a low *local* C*α* RMSD will indicate significant loop flapping. When calculating global RMSD, fitting is performed only on the V_H_ framework for the heavy-chain CDRs and on the V_L_ framework for the light-chain CDRs. This is to avoid shifts in CDR positions resulting from changes in the V_H_/V_L_ packing angle which could result from antigen binding.^18^ CDRs were defined using the AbM (Martin) loop definition^19,20^: CDR-L1 (L24–L34), CDR-L2 (L50–L56), CDR-L3 (L89–L97), CDR-H1 (H26–H35), CDR-H2 (H50–H58), CDR-H3 (H95–H102) using Chothia or Martin numbering.^16^ Structure fitting and RMSD calculation was performed using ProFit (an implementation of the McLachlan fitting algorithm^21^ available at http://www.bioinf.org.uk/software/profit/). The fitted framework region constitutes non-CDR residues, but excludes the N-terminal two residues (H1, H2, L1, L2) and the C-terminal six residues (H109–H113, L106– L110) owing to high flexibility that can lead to fitting errors and sometimes leads to missing residues in X-ray crystal structures.

### CDR canonical structure clustering

**Figure 2.**
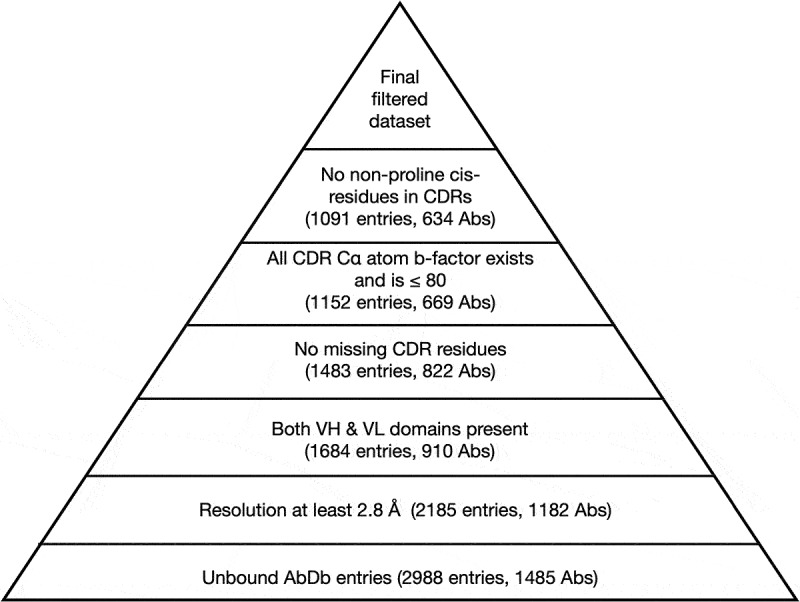
Filtering AbDb unbound structures. Filtering steps for unbound antibody structures uses the same protocol as in Figure 1. The numbers of entries and antibodies retained at each step are indicated. See supplementary file *Supp05_unbound_filtering.xlsx* for information on entries retained and rejected at each step.

Unbound CDR structures were clustered using an updated procedure based on the work of Martin and Thornton^7^ and of North *et al*.^8^ We collected *all* unbound antibodies (numbered according to the Martin scheme^16^) from AbDb with both heavy and light variable domains (V_H_/V_L_) and filtered them using the same quality criteria described in Figure 1 with the exception of the requirement for having both bound and unbound structures (and the final filtering step which relies upon having both bound and unbound structures). This led to a set of 1,091 unbound entries (Figure 2).

CDR loops were grouped based on CDR type (i.e., CDR-L1, CDR-L2, CDR-L3, CDR-H1, CDR-H2 and CDR-H3), and each group was further partitioned according to loop length and the position of any cis-proline residues. We refer to such groups as CDR ‘**L**ength and **R**esidue **C**onfiguration’ (LRC) groups. For example, the LRC group ‘L3–9-cis95’ denotes a group of CDR-L3 loops composed of 9 residues with a cis-proline at position L95.

CDR loops were then converted to vectors of sine and cosine values of dihedral angles (*φ* and *ψ*) of each residue. Each LRC group was converted to a matrix of shape *n × 4 L* where *n* denotes the number of loops, and *L* denotes the loop length. For example, a loop of length 9 (e.g. group ‘L3-9-cis95’) is converted to a 36-dimensional vector, and a set of *n* loops would be represented as an *n × 36* matrix:

$$\left[\begin{array}{c} \sin\phi_{1}^{1},\;\cos\phi_{1}^{1},\;\sin\psi_{1}^{1},\;\cos\psi_{1}^{1},\;\ldots ,\;\sin\phi_{9}^{1},\;\cos\phi_{9}^{1},\;\sin\psi_{9}^{1},\;\cos\psi_{9}^{1}\\ \ldots \\ \sin\phi_{1}^{n},\;\cos\phi_{1}^{n},\;\sin\psi_{1}^{n},\;\cos\psi_{1}^{n},\;\ldots ,\;\sin\phi_{9}^{n},\;\cos\phi_{9}^{n},\;\sin\psi_{9}^{n},\;\cos\psi_{9}^{n} \end{array}\right]$$

Each matrix was then clustered using the Affinity Propagation (AP) method.^22^ AP clustering is a message-passing-based method which has the advantage over other clustering methods of taking all data points into consideration for deciding cluster representatives. Each data point in this case is the 4*L*-element vector description of a loop as described above. The resulting clusters are referred to as ‘**AP clusters**’. The distance between a pair of loops of the same length is calculated as the squared Euclidean distance. For example, the distance between a pair of loops of the same length *L* is calculated as:

(1) $$f\left(a,b\right)={\left(\sin a-\sin b\right)}^{2}+{\left(\cos a-\cos b\right)}^{2}$$

(2) $$D(i,j)=\sum_{r=1}^{L}f(\phi_{r}^{i},\;\phi_{r}^{j})+f(\psi_{r}^{i},\;\psi_{r}^{j})$$

where *i* and *j* denote the indices of two loop conformations of interest, *r* denotes a residue index, and *L* is the loop length. The similarity between two data points (*S*(*i,j*)) is the negative squared Euclidean distance (Equation 3, below). The self-similarity *S*_*self*_, which affects the final number of clusters (as described by North *et al*.^8^) is set to the mean of similarities between all non-self pairs of CDR loops within an LRC group, i.e.,

(3) $$S\left(i,\;j\right)\;=\;-D\left(i,j\right)$$

(4) $$S_{\mathrm{self}}=\frac{2}{N(N-1)}\sum_{i=1}^{N}\sum_{j=i+1}^{N}S(i,\;j)$$

This approach to clustering is essentially the same as that of North *et al*.^8^ However, their clustering used both bound and unbound structures (as well as nonstandard antibodies), while we needed to cluster only unbound structures so that we could analyze conformational changes of CDRs on binding.

After clustering in torsional space, to decide whether a pair of AP clusters are similar in Cartesian space, we compared all possible pairs of cluster exemplars using the same criteria described by Martin and Thornton.^7^ As explained by Martin and Thornton, a difference in backbone torsion angles may correspond to a much smaller movement in Cartesian space. A pair of AP clusters is merged if their exemplar CDR structures meet all three conditions: after fitting CDRs (on C*α* atoms), the C*α* RMSD between the exemplars *<*1.0Å, the maximum distance between C*α* atoms at equivalent positions *<*1.5Å, and the maximum distance between C*β* atoms at equivalent positions *<*1.9Å. We refer to these merging criteria as the ‘*CartesianCriteria*’. The final merged AP clusters are referred to as ‘**Canonical clusters**’.

The *CartesianCriteria* were selected by Martin and Thornton to ensure the clusters were compatible with the canonical clusters described by Chothia.^6^ We ensured that this new clustering protocol was also consistent with the Chothia canonical classes (i.e., the clusters used here do not contain more than one Chothia canonical class). We also compared our canonical clusters with those obtained by North *et al*.^8^ and found the majority of class assignments were consistent given the fact that their clustering also included bound and non-V_H_/V_L_ antibodies. The methods used for these comparisons and the results are provided in Supplementary Files *Supp06_ClusterComparison.pdf* (Tables S1–S6), *Supp07_MTC_comparison.xlsx* (comparison with Martin and Thornton) and *Supp08_North_comparison.xlsx* (comparison with North *et al*.).

### Comparison of bound CDR loop conformations with unbound conformational space

**Figure 3.**
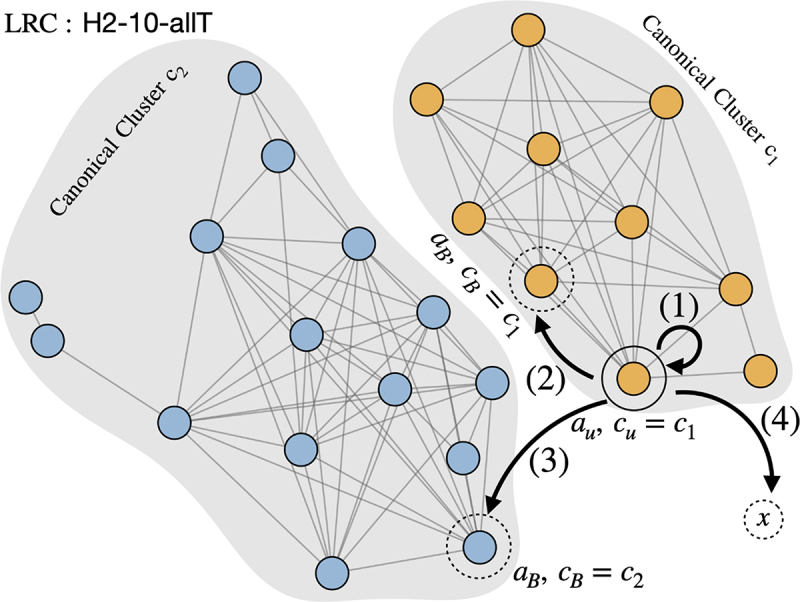
Conformational change types. The two major canonical clusters (i.e. sub-graphs) of the LRC group ‘H2–10-allT’ are shown and are denoted as *c*_1_ and *c*_2_ (area shaded in gray). Each canonical cluster consists of AP clusters (i.e., nodes in blue and orange). The AP cluster of the unbound conformer is labeled as *a*_*u*_, and its canonical cluster is denoted as *c*_*u*_, in this case, *c*_1_. The AP cluster and canonical cluster of a bound conformation *x* are denoted as *a*_*B*_ and *c*_*B*_ on the graph. Comparing *a*_*B*_,*c*_*B*_ with *a*_*u*_,*c*_*u*_, we can define four types of CDR conformational change upon binding: (1) identical AP cluster: the bound conformer is merged with the same AP cluster as the unbound. (2) AP-cluster shift: the bound conformer is merged with a different AP cluster in the same canonical cluster as the unbound. (3) canonical-cluster shift: the bound conformer is merged with an AP cluster in a canonical cluster different from the unbound; (4) non-canonical conformation: *x* is not merged with any AP cluster or canonical cluster. See Table 1.

The procedure to compare a bound CDR conformation with the unbound CDR conformational space is illustrated in Figure 3 and uses the following approach:

1. The conformational space of a CDR of a given length within an LRCgroup is represented as a set of AP clusters **A** = {*a*_1_,*a*_2_,*…,a*_*i*_} and a set of Canonical clusters **C** = {*c*_1_,*c*_2_,*…,c*_*k*_}. As a result of postcluster Cartesian merging, one canonical cluster may contain multiple AP clusters, and consequently, each AP cluster can be mapped to a single Canonical cluster.
2. A single CDR conformation is denoted as *a*^*j*^_*i*_ where *i* denotes the AP cluster and *j* denotes the conformation within that cluster. The representative (or ‘exemplar’) of an AP cluster *a*_*i*_ is denoted as *a*^*e*^_*i*_. As explained above, each *a*^*j*^_*i*_ is represented as a vector of *φ* and *ψ* sine and cosine values giving a vector size of 4 × *L* where *L* is the loop length.
3. The radius of an AP cluster *r*(*a*_*i*_) is calculated as
  (5) $$r(a_{i})=\max(D(a_{i}^{e},a_{i}^{j}))$$

where *D*() is defined in Equation 2 above. In other words, this is the maximum torsional distance between the AP cluster exemplar and any of its members.

1. The query bound conformation is transformed into a trigonometric vector as described previously, denoted as *x*.
2. For the unbound conformation of the same antibody, we identify its AP cluster *a*_*u*_ and Canonical cluster *c*_*u*_. In the example in Figure 3, representing the LRC group ‘H2-10-allT’, the unbound conformation belongs to AP cluster *a*_*u*_ in Canonical cluster *c*_*u*_, which, in this example, is *c*_1_.
3. We then locate the closest AP cluster to the bound conformation *x* (i.e., the AP cluster having the minimum value of *D*(*x,a*^*e*^_*i*_) denoted as *a*_*B*_,
4. If *D*(*x,a*_*B*_) ≤ *r*(*a*_*i*_) (i.e., the conformation falls within the radius of the cluster), then *x* is a member of AP cluster *a*_*B*_ and the associated Canonical cluster, *c*_*B*_, is identified.
5. If *D*(*x,a*_*B*_) *> r*(*a*_*i*_) (i.e., the conformation falls outside the radius of the cluster), then *x* is not a member of an existing AP cluster, but if it passes the ‘CartesianCriteria’ (defined above), then it will be a member of the Canonical cluster *c*_*B*_ of which *a*_*B*_ is a member. If it does not pass the CartesianCriteria, then conformation *x* is a novel conformation not observed in the unbound structures.

Comparing the AP cluster and Canonical cluster labels of such unbound/bound conformation pairs i.e. comparing *a*_*u*_ with *a*_*B*_ and *c*_*u*_ with *c*_*B*_, we can define four types of conformational change: 1) ‘Identical AP cluster’, 2) ‘AP-cluster shift’, 3) ‘Canonical cluster shift’, and 4) ‘Non-canonical conformation’ as described in Table 1 and Figure 3.

**Table 1.** Types of conformational change upon binding.

Type name	Definition
Identical AP cluster	The bound conformation belongs to the same AP cluster as that of its unbound conformation. i.e. there is negligible conformational change (Figure 3(1) *a*_*B*_ = *a*_*u*_, *c*_*B*_ = *c*_*u*_).
AP-cluster shift	The bound conformation belongs to a different AP cluster from that of its unbound conformation, but is within the same Canonical cluster as the unbound conformation. i.e. there is a larger conformational change in torsional space, but would be placed in the same Canonical class (Figure 3(2), *a*_*B*_ ≠ *a*_*u*_, *c*_*B*_ = *c*_*u*_).
Canonical-cluster shift	The bound conformation is different from the unbound conformation but matches a different Canonical cluster observed in another antibody (Figure 3(3), *a*_*B*_ ≠ *a*_*u*_, *c*_*B*_ ≠ *c*_*u*_, *c*_*B*_ ∈ *C*).
Non-canonical conformation	The bound conformation is different from the unbound conformation and is not seen in any other unbound the CDR unbound antibodies (Figure 3(4), *a*_*B*_ ≠ *a*_*u*_, *c*_*B*_ ≠ *c*_*u*_, *c*_*B*_ *∉ C*).

## Results

### Dataset of antibodies with unbound and bound conformers

As stated above, we use the term ‘antibody’ to refer to any set of bound or unbound structures having the same sequence and ‘entries’ to refer to the individual structures (i.e., AbDb, or AbAgDb, files). As described in the *Materials and Methods*, Figure 1 shows a schematic of the filtering procedure with the number of AbDb entries, or antibodies, retained at each stage indicated. After filtering, we identified 177 antibodies that each had at least one bound and one unbound structure from a total of 749 AbDb entries (369 unbound and 380 bound).

### CDR loop movement upon binding

**Figure 4.**
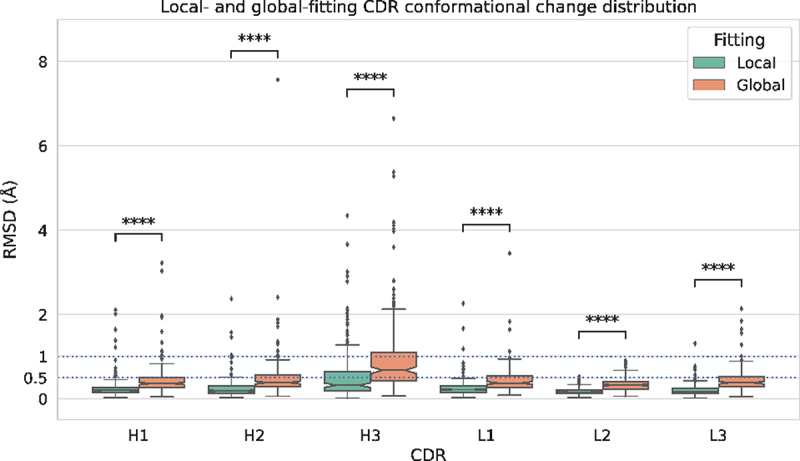
CDR conformational change distribution from global and local fitting. Distributions of conformational change (measured as C*α* RMSD) from global fitting and local fitting. Each box represents the first quartile, median and the third quartile while the whiskers represent the lower and upper fence (*Q*3 + 1.5×*IQR* meaning 3rd quartile plus one and half inter-quartile range). Outliers are shown as circles above the upper fence. To assist comparison, the C*α* RMSD at 0.5Å and 1.0Å are plotted as dashed lines. A *p*-value to compare local and global fitting was calculated using a two-sample Mann-Whitney U test. In all cases, **** indicates *p* ≤0.0001 indicating that global and local fitting are significantly different for all CDRs.

Global and local fitting were performed on all possible unbound/bound pairs of entries for each antibody, and the distribution of conformational change, as represented by the median of the C*α* RMSD for those pairs, is shown in Figure 4. For example, the mouse anti-hen egg white lysozyme antibody HyHEL-63 (PDB: 1dqq) has three associated unbound entries (1dqq_0, 1dqq_1, 1dqm_0) and three bound entries (1nbz_0P, 1dqj_0P, 1nby_0P). Thus, in this example, nine C*α* RMSD values are obtained for each CDR, from which the medians are calculated and used to plot the distribution.

As anticipated, the C*α* RMSD from local fitting (representing shape change within a loop) is consistently lower than that from global fitting. This is evident in Figure 4, which illustrates that for global fitting of non-CDR-H3 loops, the third quartile values are approximately 0.5Å. Over 70% of non-CDR-H3 loops (with the exception of CDR-H2 at 68%) exhibit a global C*α* RMSD of less than 0.5Å, as detailed in Table 2(1). A global C*α* RMSD of up to 0.5Å is commonly regarded as a typical level of error in crystal structures^23^ and other work suggests that the difference seen in multiple structures of the same protein crystallized under varying conditions and in different space groups can be as high as 1.2Å.^24^ Thus, 0.5Å is a very conservative value and our finding that the majority of non-CDR-H3 loops undergo movements of ≤ 0.5Å clearly implies that the CDRs typically exhibit minimal backbone movement upon binding.

**Table 2.** Numbers of antibodies in different **C***α* RMSD ranges.

(1) Global fitting C*α* RMSD (Å)						
CDR	≤0.5	(0.5,1.0]	(1.0,2.0]	(2.0,3.0]	(3.0,4.0]	*>* 4.0
H1	131 (74%)	36 (20%)	8 (5%)	0	2 (1%)	0
H2	120 (68%)	46 (26%)	9 (5%)	1 (1%)	0	1 (1%)
H3	60 (34%)	65 (37%)	29 (16%)	15 (8%)	2 (1%)	6 (3%)
L1	127 (72%)	46 (26%)	3 (2%)	0	1 (1%)	0
L2	155 (88%)	22 (12%)	0	0	0	0
L3	130 (73%)	42 (24%)	4 (2%)	1 (1%)	0	0
(2) Local fitting C*α* RMSD (Å)						
CDR	≤0.5	(0.5,1.0]	(1.0,2.0]	(2.0,3.0]	(3.0,4.0]	*>* 4.0
H1	163 (92%)	9 (5%)	3 (2%)	2 (1%)	0	0
H2	166 (94%)	7 (4%)	3 (2%)	1 (1%)	0	0
H3	120 (68%)	28 (16%)	20 (11%)	6 (3%)	2 (1%)	1 (1%)
L1	167 (94%)	7 (4%)	2 (1%)	1 (1%)	0	0
L2	176 (99%)	1 (1%)	0	0	0	0
L3	172 (97%)	4 (2%)	1 (1%)	0	0	0
(3) Diference between global and local fitting C*α* RMSD (Å)						
CDR	≤0.25	(0.25,0.5]	(0.5,1.0]	(1.0,2.0]	(2.0,3.0]	*>* 3.0
H1	138 (78%)	32 (18%)	6 (3%)	1 (1%)	0	0
H2	122 (69%)	44 (25%)	9 (5%)	1 (1%)	0	1 (1%)
H3	72 (41%)	57 (32%)	33 (19%)	12 (7%)	3 (2%)	0
L1	141 (80%)	33 (19%)	2 (1%)	1 (1%)	0	0
L2	153 (86%)	22 (12%)	2 (1%)	0	0	0
L3	122 (69%)	44 (25%)	8 (5%)	3 (2%)	0	0

The percentage of each count relative to the entire dataset (177 antibodies) is given in parentheses. The difference between global and local fitting C*α* RMSD, which reflects loop flapping, is calculated as the absolute value of the difference between the global and local C*α* RMSD of each pair of unbound/bound entries.

Meanwhile, when locally fitted, the third quartiles for non-CDR-H3 loops dropped to 0.25Å (Figure 4), and over 90% of non-CDR-H3 loops showed a local fitting C*α* RMSD below 0.5Å (Table 2(2)). Comparing global and local fitting, we observed the average percentage of antibodies with a CDR having C*α* RMSD below 0.5Å increased from 68.2% (average of percentages in Table 2(1), column 1) to 90.7% (average of percentages in Table 2(2), column 1) and those within the range of 0.5Å and 1.0Å dropped from 24.2% to 5.3% (average of column 2 percentages in Table 2(1) and Table 2(2), respectively). A two-sample Mann-Whitney U test was conducted to compare the global and local C*α* RMSD for each CDR loop and found to be significant at *p* ≤0.0001 in all cases (Figure 4). This clearly demonstrates that part of the global C*α* RMSD is caused by a small degree of loop ‘flapping’.^17^ We also calculated the difference between global and local C*α* RMSD for each antibody as an indicator of the amount of loop flapping (Table 2(3)). Generally, we observed an average of 70.5% of CDRs in antibodies that showed a difference of up to 0.25Å (average of percentages in Table 2(3), column 1; i.e., no loop flapping) and 21.8% of antibodies between 0.25Å and 0.5Å (average of percentages in Table 2(3), column 2; i.e., minimal flapping).

The exception is CDR-H3 with a box-plot upper fence value (see legend to Figure 4) of 1.31Å from local fitting (Figure 4). However, this is still lower than the upper fence value of 2.14Å from global fitting. The percentage of antibodies showing a C*α* RMSD below 0.5Å increases from 34% for global fitting to 68% for local fitting (CDR-H3 in column 1 of Table 2(1) compared with Table 2(2)). Thus, CDR-H3 more frequently shows larger scale flapping movements than the other CDRs. 19% of CDR-H3 loops showed a C*α* RMSD difference (local *vs*. global) between 0.5Å and 1.0Å, whereas this value was ≤ 5% for non-CDR-H3 loops (CDR-H3 in Table 2(3), column 3). Thus, loop ‘flapping’ is more common in CDR-H3 upon binding than in non-CDR-H3 loops.

The findings for CDR-H3 and non-CDR-H3 loops at cutoffs of ≤ 1.0Å and ≤ 2.0Å are summarized in Table 3. Most (96.7%) non-CDR-H3 loops show a global fit with a C*α* RMSD of ≤ 1.0Å, while almost all (99.3%) show a global fit of ≤ 2.0Å. The local fitting values rise to 98.5% and 99.5%, respectively. This suggests that non-CDR-H3 loops rarely change conformation on binding. Further, the fact that the percentage of non-CDR-H3 loops with local and global C*α* RMSD ≤ 2.0Å is virtually unchanged suggests that while some loop flapping occurs, it is only a small effect (mostly ≤1.0Å).

**Table 3.** Summary of global and local C*α* fitting for non-CDR-H3 loops and CDR-H3 loops.

	≤1.0Å		≤2.0Å	
	Non-H3	H3	Non-H3	H3
Global	96.7%	70.6%	99.3%	87.0%
Local	98.5%	83.6%	99.5%	94.9%

The percentages of loops having C*α* RMSD values ≤ 1.0Å and ≤ 2.0Å are shown.

On the other hand, 70.6% of CDR-H3 loops show a global fit with a C*α* RMSD of ≤ 1.0Å, while 87.0% show a global fit of ≤ 2.0Å. The local fitting values rise to 83.6% and 94.9%, respectively. This suggests that while changes in CDR-H3 conformation on binding are still uncommon, they are much more common than for the non-CDR-H3 loops. The fact that ∼8% and ∼13% more of the CDR-H3 loops have local C*α* RMSD of ≤ 2.0Å and ≤ 1.0Å, respectively, suggests both that loop flapping is much more common in CDR-H3 than it is in the other CDRs and that the degree of flapping is greater than with the other CDRs.

**Figure 5.**
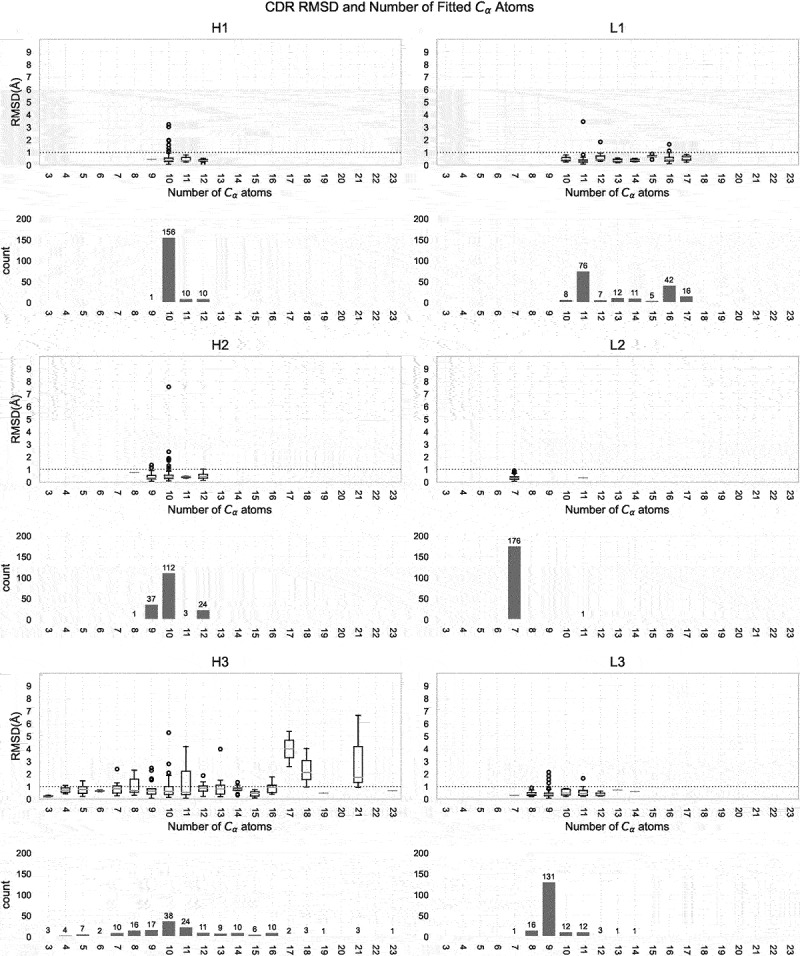
CDR loop movement upon complexation against loop length. The global C*α* RMSD of each CDR upon binding (C*α* RMSD) versus loop length (number of residues) are plotted as boxplots, with outliers (exceeding upper fence values *Q*3 + 1.5×*IQR*) shown as circles. A horizontal dashed line is drawn at 1.0Å C*α* RMSD on each box plot. The number of CDR loops of each loop length are also plotted as histograms.

CDR conformational change from global fitting was also plotted against loop length (Figure 5). A single loop length group dominates CDR-H1, CDR-H2, CDR-L2, and CDR-L3. In our dataset, CDR-L1 has two major groups: 11 and 16 residues. In contrast, CDR-H3 has diverse loop lengths, with the majority being between 7 and 16 residues. For CDR-H3 loops, little correlation between conformational change and loop length was observed (Spearman rank correlation coefficient between global C*α* RMSD and loop length is 0.13; *p*-value of 0.08). However, we do observe a larger conformational change when the loop becomes longer for ten antibodies with CDR-H3 loop length ≥17 residues: the CDR-H3 global C*α* RMSD from such antibodies ranges between 0.93Å and 6.65Å, see Figure 5(H3). Although it appears that the longer loops might commonly undergo a larger conformational change upon binding, this may be a result of the limited number of antibodies with such long CDR-H3 loops (only 10 antibodies in AbAgDb have a CDR-H3 loop longer than 16 residues which only accounts for 7% of entries).

### CDR conformational clustering

**Figure 6.**
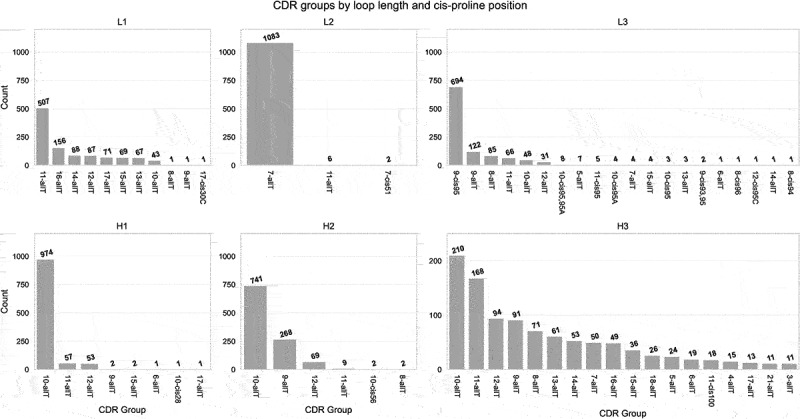
LRC groups. Each subplot shows the number of entries in each LRC group. For CDR-H3, only groups with more than 10 entries are shown.

The LRC distribution of each CDR is shown in Figure 6 sorted by group size. Both CDR-L2 and CDR-H1 are dominated by a single group. ‘L2–7-allT’ accounts for 99% of entries for CDR-L2, while ‘H1–10-allT’ accounts for 89% of entries for CDR-H1 where the second biggest group (‘H1–11-allT’) only represents 5%. CDR-H2 is dominated by ‘H2–10-allT’ accounting for 68% of entries followed by two smaller groups, ‘H2–9-allT’ (25%) and ‘H2–12-allT’ (6%). CDR-L1 is dominated by ‘L1–11-allT’ (46%) followed by ‘L1–16-allT’ (14%) and six smaller groups each of which accounts for less than 8% of entries. Similarly, CDR-L3 has a single dominant group (‘L3–9-cis95’, 64%) followed by ‘L3–9-allT’ (11%) and four smaller groups each representing up to 8%, with the rest being much less well populated.

The same descriptor was applied to CDR-H3 which consists of numerous small groups – the two most common LRC groups (‘H3–10-allT’ and ‘H3-11-allT’) account for 19% and 15% of entries, respectively, while six groups each represents 5–9% of entries. The rest of the CDR-H3 LRC groups are much less common.

**Figure 7.**
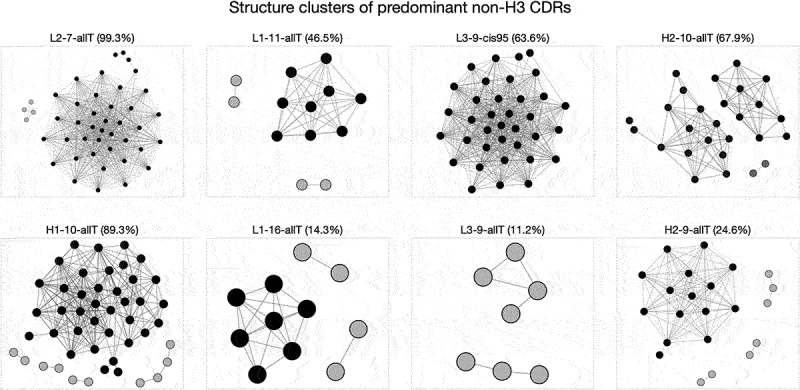
Structure clusters of predominant non-CDR-H3 CDR LRC groups. Subplot titles are CDR LRC group names, and the percentage given in parenthesis denotes the ratio of the group size (number of AbDb entries) to the entire set (1091 entries). Each node represents an AP cluster which consists of a set of similar CDR structures and from which a representative structure (also called an exemplar structure) was identified. The edges between pairs of nodes indicate the exemplar structures of both nodes are similar following our criteria under Cartesian space and thus belong to the same canonical cluster. Nodes, directly or indirectly connected, are given the same color. The major groups are colored in black and smaller ones in gray. Note we use edges to indicate connectivity only, which means the distance between a pair of nodes is trivial in this case. The placing of nodes in the figure is purely illustrative.

To derive a representation of the unbound CDR conformational space, we performed torsional clustering within each LRC group to generate ‘AP clusters’. As an example, Figure 7 shows the clustering results for the largest LRC groups of each CDR. Groups including ‘L2–7-allT’, ‘H1–10-allT’, ‘L1-11allT’, ‘L1–16-allT’ and ‘H2–9-allT’ comprise a leading conformational cluster with a few smaller clusters. Groups including ‘H2–10-allT’ and ‘L3–9-allT’ are composed of two leading conformational clusters, and ‘H2–10-allT’ has an additional small cluster. Group ‘L3–9-cis95’ is dominated by a single conformational cluster. The contents of each cluster (including the CDR sequences) are shown in Supplementary File *‘Supp09_cluster_member_cdr_seq.xlsx’*. After torsional clustering, we performed Cartesian cluster merging to replicate the Chothia canonical clusters as described by Martin and Thornton.^7^

Although CDR-H3 does not follow the canonical class rules adopted by the other CDRs, we clustered the observed LRC groups for CDR-H3 in the same way, forming AP (torsional) clusters and then ‘canonical’ clusters by Cartesian cluster merging.

### CDR conformational change types

The numbers of antibodies of each conformational change type (as described in Table 1) are summarized in Table 4. For all CDRs except CDR-H3, 98–100% of bound conformations are observed in unbound antibodies (Table 4, column ‘Sum(NR)’). While some degree of conformational change at the torsional level is observed frequently (40–74% of the time; Table 4 column ‘AP ClusterShift’), large changes are rare (1–3% change to a different canonical cluster; 0–2% to a conformation not seen as part of a canonical cluster in unbound antibodies).

**Table 4.** Counts of antibodies of each conformational change type.

	Unbound conformational space				
CDR	Identical-AP	AP-cluster shift	Canonical cluster shift	Sum (NR)	Non-canonical conformation
H1	127 (72%)	95 (54%)	3 (2%)	174 (98%)	3 (2%)
H2	123 (69%)	89 (50%)	3 (2%)	174 (98%)	4 (2%)
H3	138 (78%)	6 (3%)	21 (12%)	154 (87%)	19 (11%)
L1	144 (81%)	70 (40%)	5 (3%)	175 (99%)	3 (2%)
L2	87 (49%)	131 (74%)	1 (1%)	177 (100%)	0 (0%)
L3	114 (64%)	91 (51%)	2 (1%)	175 (99%)	3 (2%)

Because one antibody can have more than one unbound and/or bound entries, it can fall into multiple conformational change types and therefore the total number of cases from the four types can exceed the number of antibodies in the entire set (177 antibodies). **Sum (NR)** is the sum of non-redundant antibodies whose bound conformation can be found in the unbound conformational space (‘Identical-AP’, ‘AP-cluster shift’, ‘Canonical-cluster shift’).

In contrast, for CDR-H3, only 87% of bound conformations can be found in the unbound conformational space, as indicated in the ‘Sum(NR)’ column of Table 4. CDR-H3 loops also exhibit a higher occurrence of ‘Canonical-cluster shift’ and ‘Non-canonical conformation’, with proportions of 12% and 11%, respectively, as shown in the corresponding columns of Table 4. To assess the statistical significance of these differences, we performed three *χ*^2^ tests. Initially, we performed a 6 × 2 test on the data in Table 4 (CDR-L1–CDR-H3 *vs*. ‘Sum(NR)’/‘Non-canonical conformation’) which showed significant differences (*p <* 1 × 10^−4^). However, as CDR-H3 appeared to be the only CDR to show any major differences, we removed CDR-H3 from the analysis and performed a 5 × 2 test on the data (CDR-L1–CDR-H2 *vs*. ‘Sum(NR)’/‘Non-canonical conformation’) which showed no significant difference within the non-CDR-H3 loops (*p* = 0.467). Finally, we also performed a 6 × 2 test (CDR-H3/non-CDR-H3 *vs*. ‘Sum(NR)’/‘Non-canonical conformation’) which confirmed that the increased movement to non-canonical conformations in CDR-H3 is significant (*p* ≪ 1 × 10^−4^).

**Figure 8.**
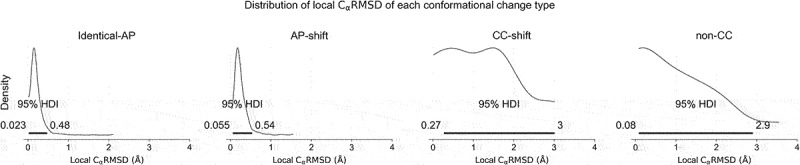
Local C*α* RMSD of antibodies of each conformational change type. Each subplot is a kernel density estimation of the local C*α* RMSD of unbound/bound CDR conformation pairs found in each conformational change type. The number in each subplot parenthesis indicates number of antibodies.

**Figure 9.**
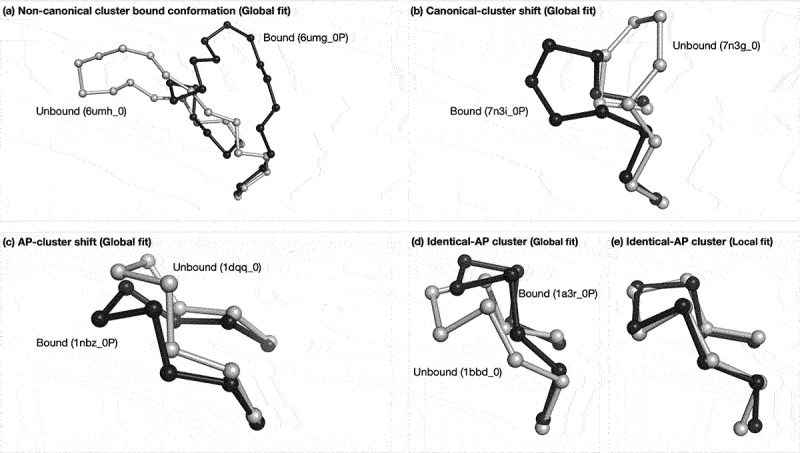
Example of each conformational change type. The figure shows one locally fitted CDR-H3 unbound/bound pair for each conformational change type. The bound CDR-H3 loop is colored black and the unbound loop is gray. (a) Non-canonical cluster conformation: unbound (6umh_0) and bound (6umg_0P), global C*α* RMSD of 8.41Å; (b) Canonical-cluster shift: unbound (7n3g_0) and bound (7n3i_0P), global C*α* RMSD of 5.28Å; (c) AP-cluster shift: unbound (1kcv_0) and bound (1kcs_0P), global C*α* RMSD of 2.38Å; (d) and (e) identical-AP cluster — (d) is locally fitted and (e) is globally fitted to show the loop ‘flapping’ effect. (d) global C*α* RMSD 2.27Å, (e) global C*α* RMSD 0.54Å.

In addition, we plotted the density distribution of local C*α* RMSD for antibodies of each conformational change type (Figure 8). Generally, the conformational change for CDRs of ‘Identical-AP’ and ‘AP-cluster shift’ conformational change type is minimal (around 0.5Å), whereas those of ‘Canonical cluster shift’ and ‘Non-canonical conformation’ types are larger and more wide-ranging. Examples of unbound/bound pairs for each conformational change type are provided in Figure 9, and the loop ‘flapping’ effect is evident in Figure 9d,e where the local C*α* RMSD is small and much lower than the global C*α* RMSD.

### Effect of differences in antigens on CDR conformation

While affinity-matured antibodies generally have high specificity as well as high affinity, there are examples of such antibodies that bind to mutant (or, in rare cases, different) antigens. For example, structures have been solved of anti-hen egg white lysozyme (EWL) antibodies HyHEL-5 bound to bobwhite quail EWL (1bql); HyHEL-10 bound to Pekin duck EWL (5fjo); and both HyHEL-10 (6p4a) and HyHEL-16 (1nbz, 1dqj, 1nby) bound to hen EWL mutants. Consequently, it is possible that CDR movement may occur when bound to a mutant, but not to an antigen against which the antibody has been raised (or *vice versa*). Movements in binding different antigens have been observed previously.^13^

**Figure 10.**
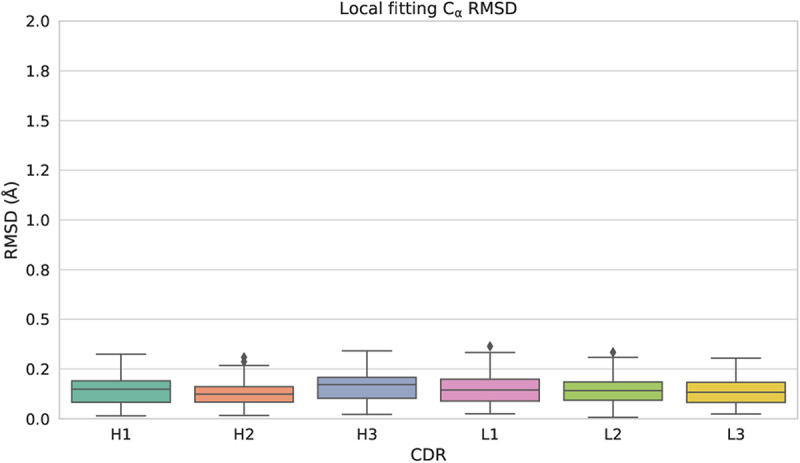
Impact of antigen mutations on CDR conformation. CDRs within clusters of identical antibodies binding to multiple antigens were locally fitted with one another and the C*α* RMSDs were plotted. Where, within this set, there was more than one example of the same antibody binding to the same antigen, an exemplar was selected.

Initially, we identified antibodies binding to multiple antigens with sequence identities above 70% to one another using CD-HIT. We then calculated the pairwise local C*α* RMSD between the equivalent CDR loops in each cluster. Figure 10 shows that the majority of pairwise C*α* RMSD values are below 0.5Å, indicating a minimal effect of antigen mutations on CDR conformations, at least in this dataset of similar antigens.

## Discussion

It is sometimes suggested, particularly by those who have not studied antibodies in detail, that CDRs may undergo significant conformational change when binding to an antigen. If this were true, it would call into question the use of modeling, or crystallography, of unbound structures to make predictions about the bound form. However, from a thermodynamic perspective, given all other things being equal, a rigid ‘lock-and-key’ interaction will result in optimal affinity with no loss of enthalpy or entropy. Consequently, in this work, we provide a survey of CDR conformational change upon binding by directly comparing the unbound and bound conformers of the same antibody.

We implemented a filtering pipeline to pool high-quality antibody structures from AbDb^14^ and built a primary dataset (AbAgDb) consisting of 177 antibodies with bound and unbound structures. When examining any type of movement in proteins, it is possible that filtering out lower-quality structures (those with poor resolution, missing residues or high temperature factors) may result in discarding structures that are flexible (because the flexibility leads to poorer crystals and therefore lower resolutions, or residues that cannot be seen in the electron density map). On the other hand, when looking at differences in lower-quality structures, it is impossible to know whether these differences are real, or simply an artifact of the poor-quality structure. It is well known that NMR structures give a better picture of the flexibility of a protein in solution rather than the snapshot shown by X-ray crystallography, but, as described above, in the work performed here, there were no examples of NMR structures of antibodies where both bound and unbound versions were available.

It was our aim to consider CDR conformational changes on binding, not to look at flexibility within the unbound structure. Consequently, while it is possible that we are eliminating examples of the third model for protein–protein interactions (conformational-selection), we are exploring the other two models for binding (lock-and-key and induced-fit) with a high level of confidence. It should be noted that the filtering criteria that we used are common practices for selecting high quality structures generally used in structural analysis and the numbers of structures removed are small. In the case of non-prolines adopting a cis peptide bond, genuine examples are extremely rare.^25^ When they do occur, they tend to be in regions of functionally important steric strain^26,27^ (unlikely in a CDR loop) and they tend to occur in particular types of proteins.^25^ Indeed, Williams *et al*.^25^ have emphasized the importance of very strict quality filtering when trying to identify genuine cis non-prolines. Consequently, while we may be eliminating examples of conformational selection, this filtering is a prudent approach and the number of structures removed is small.

To explore whether this has been detrimental to our analysis, we used an auxiliary dataset of antibodies that had been filtered-out and rejected from the primary dataset based on high B-factors or poor resolution, and identified those with identical CDRs to antibodies in the AbAgDb primary dataset. We did not find any significant differences in conformational change on binding between the primary and the auxiliary datasets (*p >* 0.1 in all cases, two-sample Mann-Whitney U test). (Supplementary File *Supp10_PrimaryAuxiliaryComparison.pdf*, Figure S1 and Table S1; Supplementary File *Supp11_auxiliary_set.xlsx*). We also explored whether the filtered-out antibodies in the auxiliary dataset have a higher scale of CDR conformational change by comparing the CDR conformational change distribution between the primary and auxiliary datasets. In general, the CDR conformational change distribution is larger in the auxiliary dataset for both local and global fitting (*p <* 1 × 10^−4^, two-sample Mann-Whitney U test), except for CDR-H3 loops when fitted globally (*p* = 0.9838), implying similar CDR conformational change scale of CDR-H3 loops between the two datasets (Supplementary File *Supp10_PrimaryAuxiliaryComparison.pdf*, Figure S2 and Table S2). While there is generally a significant difference, it is impossible to know whether this difference in scale is a real effect (resulting from differences in flexibility), or simply poor-quality data. It should also be noted that four antibodies were removed from the analysis where the frameworks showed ≥1.0Å C*α* RMSD between bound and unbound versions. This was done to avoid misleading the analysis of local *vs*. global RMSD within the CDRs, but these are clearly cases where there is some substantial degree of conformational change on binding that affects the framework as well as the CDRs. However, this removed only four antibodies from the analysis.

Currently, our dataset is confined to structures of conventional variable fragments (Fvs) containing both V_H_ and V_L_ domains. Compared with a previously published dataset for antibody-antigen structures,^28^ our dataset has expanded the number of antigen types and examples. We believe that maintaining this dataset is beneficial for the development of new computational tools for antibody-related tasks, such as epitope prediction and antibody-antigen complex prediction. As reviewed recently,^2^ one of the major challenges in developing computational tools for antibody development is data completeness.

We investigated the conformational changes of each CDR loop using global and local fitting while excluding changes resulting from differences in the packing of V_H_ and V_L_ domains. In summary, the local C*α* conformation of CDRs other than CDR-H3 changes by ≤ 1.0Å in 98.5% of cases and by ≤ 2.0Å in 99.5% of cases, indicating that large conformational changes are rare. In CDR-H3, these percentages drop to 83.6% (≤1.0Å) and 94.9% (≤2.0Å) indicating that conformational change is more common, but still unusual. See Table 3.

However, when we look at the *global* C*α* RMSD, we find that smaller percentages of all CDRs have C*α* RMSD below either 1.0Å or 2.0Å, indicating loop flapping. For the non-CDR-H3 loops, the global and local percentages are almost the same when looking at RMSDs ≤ 2.0Å, indicating only a minor flapping effect, but this is much more frequent in CDR-H3 (Table 3). Our findings agree with early work by Bajorath *et al*.^17^ who studied just seven antibody structures (two bound and five unbound) and found that local fitting generally showed a C*α* RMSD of up to 0.5Å while global fitting showed 1.5 − 2.7Å.

We went on to cluster unbound CDR conformations in backbone torsion angles to create ‘AP clusters’ followed by Cartesian cluster merging to create ‘Canonical clusters’. This approach was applied to all six CDRs. We then classified the conformational change on binding into four categories: ‘identical AP cluster’, ‘AP-cluster shift’, ‘canonical-cluster shift’, and ‘non-canonical structure’, as described in Table 1.

In most cases, CDR conformation does not change on binding, at least at the level of a canonical cluster. Specifically, for non-CDR-H3 loops, approximately 1–3% undergo a change in canonical cluster, and 0–2% change to a conformation not observed in canonical clusters of unbound antibodies (Table 4). While CDR-H3 loops are more likely to change conformation (either adopting non-canonical conformations or going through canonical cluster shifts) than the other CDR loops, the vast majority (87%, ‘Sum(NR)’ Table 4) of bound CDR-H3 loops that can be found in the unbound conformational space. Only around 12% of CDR-H3 loops shift to a canonical cluster observed in other unbound antibodies, while 11% adopt conformations not seen in unbound antibodies (Table 4).

Canonical class shifts are rare, but when we do see them, they are all changes to conformations seen in a different antibody, with the exception of three antibodies in which the CDR-H3 loops change to a conformation seen in a different entry for the same antibody, indicating flexibility in the CDR-H3 of these three antibodies.

In conclusion, the notion that antibody CDRs go through significant conformational change upon binding to an antigen is not supported by our work. Instead, we show here that, while this does occur (particularly in CDR-H3), it is uncommon. We provide a survey of CDR movement, directly comparing the unbound and bound conformers of the same antibody, both by C*α* RMSD and by conformational clustering. Based on our AbAgDb dataset of 177 high-quality antibody structures where both unbound and bound forms are available, we found that significant local conformational change on binding is rare. Only ∼1.5% show a local conformational change of *>*1.0Å (C*α* RMSD) and ∼0.5% show a local conformational change of *>*2.0Å. Conformational change is somewhat more common in CDR-H3, but most antibodies still undergo only minimal change in CDR-H3 (∼16.4% show a local conformational change of *>*1.0Å, while only ∼5.1% show a local conformational change of *>*2.0Å). We also observed a loop ‘flapping’ effect where there is minimal change in CDR conformation, but the loop ‘flaps’ about its junction with the framework, agreeing with previous work on a very small dataset.^17^ This was found always to be a minor effect in non-CDR-H3 loops, but is somewhat more common and larger in CDR-H3.

## Availability

- A snapshot of AbDb (version date: 20220926) as used to build AbAgDb is available at http://www.abybank.org/abdb/snapshots/abdb_20220926.zip
- ProFit was used for protein structure fitting and can be obtained from http://www.bioinf.org.uk/software/profit/
- The code for using the CDR conformation classifiers is available at https://github.com/biochunan/CDRConformationClassification
- Supplementary File *Supp00_README.txt* details the content and format of the other supplementary files.

## Supplementary Material

Supplemental Material
