# Supplementary material for: Do antibody CDR loops change conformation upon binding?
Source: MAbs. 2024 Mar 13;16(1):2322533. doi: 10.1080/19420862.2024.2322533 (PMC10939163; doi:10.1080/19420862.2024.2322533)
Supplement: Supplemental Material [file KMAB_A_2322533_SM0152.zip › Supp06_ClusterComparison--.docx]

Do antibody CDR loops change conformation upon binding?

**Supplementary Material**

Chu’nan Liu, Lilian M. Denzler,
Oliver E.C. Hood, and Andrew C.R. Martin

Structural and Molecular Biology, Division of Biosciences

University College London

Gower Street, London WC1E 6BT

February 7, 2024

# Clustering comparison with Martin and Thornton (1996)

We compared the clustering results in this work with the clustering of 48 antibody structures described in Martin and Thornton (1996)^^[[1]](#footnote-1)^^, primarily to ensure that the clusters generated here were compatible with the canonical classes defined by Chothia and Lesk.

Clustering results are provided in the following Tables S1–S6.

The 48 antibodies used in Martin and Thornton (1996) were a mixture of bound and unbound structures. Of these, 23 were unbound and thus found in our dataset while the remaining 25 were structures of the antibody-antigen complex. Two of the bound structures, ‘1jel’ and ‘2hfl’ are now obsolete and have been superseded by ‘2jel’ and ‘1yqv’ respectively. Consequently, AbDb entries ‘2jel_0P’ and ‘1yqv_0P’ were used in this comparison.

For the 23 unbound structures, the clustering results were obtained from the analysis described in this paper. Each of the remaining 25 bound antibodies were assigned to clusters as follows:

- the length of the CDR was determined and all LRC groups having that length were identified.
- the CDR conformation was converted to the trigonometric representation as described in the *Materials and Methods*.
- this representation was then compared against all AP clusters within all LRC groups of the required length. e.g. CDR-L3 of PDB file 1bbd, (AbDb entry: 1bbd0) has a length of 9 residues; there are two LRC groups of length 9 (‘L3-9-cis95’ and ‘L3-9-allT’) so all AP clusters in those two LRC groups were used.
- the process described in the *Materials and Methods* was then used to decide whether this CDR can be assigned to an AP cluster.
- If the CDR *is* assigned to an AP cluster, then the corresponding LRC group, AP cluster (represented by the cluster exemplar AbDb entry name), and the canonical cluster index are reported. In Tables S1–S5, these are reported in columns **‘LRC’**, **‘AP’** and **‘CAN’** respectively.
- If the CDR *is not* assigned to an AP cluster, these values are denoted as a dot (‘ . ’) in the tables, indicating the query CDR conformation does not match any of the canonical conformations identified in the unbound antibodies.

Tables S1–S5 are shaded with alternating colours to distinguish loop length and LRC groups. Tables S1–S5 also show the relevant results from Table 6 in Martin and Thornton (1996): structural clusters and canonical class assignments based on key residues defined by Chothia and Lesk. These appear in columns **‘M&T1996’** and **‘Chothia’** respectively. ‘?’ in the **‘Chothia’** column indicates loops that could not be assigned to a canonical class using Chothia and Lesk’s key residues. The same data are provided in Supplementary File *Supp07_MTC comparison.xlsx*.

In performing the current work, two errors were identified in Table 6 of Martin and Thornton (1996):

- For CDR-H1, 1ggi was labelled as unknown Chothia class (‘?’) and structural cluster 10A instead of class 3 and cluster 12A respectively.
- For CDR-H2, 1ncb was labelled as Chothia class 4 instead of class 2.

These errors are corrected in Tables S4 and S5 below and in Supplementary File *Supp07_MTC_comparison.xlsx*.

The results in Tables S1–S5 demonstrate that the clustering results provided here are compatible with Chothia and Lesk’s analysis. Specifically, no cluster defined in this work is a superset of multiple Chothia canonicals. However, as with the Martin and Thornton (1996) analysis, there are some cases where the clustering performed here identifies multiple clusters that would be assigned to a single Chothia canonical on the basis of key residues that they have defined.

Table S1: Clustering results for CDR-L1 loops.

| **AbDb ID** | **LRC** | **AP** | **CAN** | **Chothia** | **M&T1996** |
| --- | --- | --- | --- | --- | --- |
| 1for_0 | L1-10-allT | 2w9d_0 | 0 | 1 | 10A |
| 1yqv_0P (2hfl) | L1-10-allT | 2w9d_0 | 0 | 1 | 10A |
| 1baf_0H | L1-10-allT | 4ebq_0 | 0 | 1 | 10A |
| 2fbj_0H | L1-10-allT | 4ebq_0 | 0 | 1 | 10A |
| 1dfb_0 | L1-11-allT | 6n35_0 | 0 | 2 | 11A |
| 1fai_0 | L1-11-allT | 1zan_0 | 0 | 2 | 11A |
| 1fvc_0 | L1-11-allT | 3hc4_0 | 0 | ? | 11A |
| 1fvd_0 | L1-11-allT | 4fnl_1 | 0 | ? | 11A |
| 1igc_0 | L1-11-allT | 3hc4_0 | 0 | ? | 11A |
| 1igm_0 | L1-11-allT | 3hc4_0 | 0 | 2 | 11A |
| 1mam_0 | L1-11-allT | 6i1o_0 | 0 | 2 | 11A |
| 1mlb_0 | L1-11-allT | 6n35_0 | 0 | 2 | 11A |
| 1vfa_0 | L1-11-allT | 1zan_0 | 0 | 2 | 11A |
| 6fab_0 | L1-11-allT | 6i1o_0 | 0 | 2 | 11A |
| 1bbj_1 | L1-11-allT | 5i8e_0 | 0 | 2 | 11A |
| 2gfb_4 | L1-11-allT | 1zan_0 | 0 | 2 | 11A |
| 1fbi_1P | L1-11-allT | 1zan_0 | 0 | 2 | 11A |
| 1ikf_0P | L1-11-allT | 1zan_0 | 0 | 2 | 11A |
| 1jhl_0P | L1-11-allT | 1zan_0 | 0 | 2 | 11A |
| 1ncb_0P | L1-11-allT | 1zan_0 | 0 | ? | 11A |
| 3hfm_0P | L1-11-allT | 1zan_0 | 0 | 2 | 11A |
| 1eap_0H | L1-11-allT | 6i1o_0 | 0 | ? | 11A |
| 8fab_0 | L1-11-allT | 8fab_0 | 8 | ? | 11B |
| 1fig_0H | . | . | . | 6 | 12A |
| 2fb4_0 | L1-13-allT | 4q2z_0 | 1 | 5λ | 13A |
| 1gig_0 | L1-14-allT | 3cfj_3 | 0 | 7λ | 14B |
| 1ind_0H | L1-14-allT | 3cfj_3 | 0 | 7λ | 14B |
| 7fab_0 | L1-14-allT | 7mu4_0 | 4 | 6λ | 14A |
| 1mfa_0H | . | . | . | 7λ | 14B |
| 1acy_0P | L1-15-allT | 2vl5_1 | 0 | ? | 15B |
| 1ibg_0H | L1-15-allT | 1mf2_0 | 0 | ? | 15B |
| 1ggi_0P | . | . | . | 5 | 15A |
| 1cgs_0 | L1-16-allT | 1cgs_0 | 2 | ? | 16A |
| 1igi_0 | L1-16-allT | 4jn1_0 | 2 | 4 | 16A |
| 1lmk_0 | L1-16-allT | 1jgv_0 | 2 | ? | 16A |
| 1rmf_0 | L1-16-allT | 4jn1_0 | 2 | 4 | 16A |
| 2jel_0P (1jel) | L1-16-allT | 1jgv_0 | 2 | ? | 16C |
| 1fpt_0P | L1-16-allT | 1jgv_0 | 2 | 4 | 16A |
| 1dbb_0H | L1-16-allT | 1cgs_0 | 2 | 4 | 16A |
| 1tet_0PH | L1-16-allT | 7bg1_0 | 2 | ? | 16A |
| 2cgr_0H | L1-16-allT | 1jgv_0 | 2 | ? | 16A |
| 1igf_0 | L1-16-allT | 1igf_0 | 3 | ? | 16A |
| 1nbv_0 | L1-16-allT | 1nbv_0 | 4 | 4 | 16B |
| 4fab_0H | . | . | . | 4 | 16B |
| 1bbd_0 | L1-17-allT | 3ijh_1 | 0 | 3 | 17A |
| 1hil_0 | L1-17-allT | 3ijh_1 | 0 | 3 | 17A |
| 1mcp_0 | L1-17-allT | 1mcp_0 | 0 | 3 | 17A |
| 1frg_0P | L1-17-allT | 3ijh_1 | 0 | 3 | 17A |

Table S2: Clustering results for CDR-L2 loops.

| **AbDb ID** | **LRC** | **AP** | **CAN** | **Chothia** | **M&T1996** |
| --- | --- | --- | --- | --- | --- |
| 1bbd_0 | L2-7-allT | 5fcs_1 | 0 | 1 | 7A |
| 1cgs_0 | L2-7-allT | 1bln_1 | 0 | 1 | 7A |
| 1dfb_0 | L2-7-allT | 1bln_1 | 0 | 1 | 7A |
| 1fai_0 | L2-7-allT | 6elj_0 | 0 | 1 | 7A |
| 1for_0 | L2-7-allT | 1a7n_0 | 0 | 1 | 7A |
| 1fvc_0 | L2-7-allT | 6j9o_0 | 0 | 1 | 7A |
| 1fvd_0 | L2-7-allT | 5bvj_3 | 0 | 1 | 7A |
| 1gig_0 | L2-7-allT | 3v0v_0 | 0 | 1 | 7A |
| 1hil_0 | L2-7-allT | 5gs1_2 | 0 | 1 | 7A |
| 1igc_0 | L2-7-allT | 1etz_0 | 0 | 1 | 7A |
| 1igf_0 | L2-7-allT | 1a7n_0 | 0 | 1 | 7A |
| 1igi_0 | L2-7-allT | 5bvj_3 | 0 | 1 | 7A |
| 1igm_0 | L2-7-allT | 6btj_0 | 0 | 1 | 7A |
| 1lmk_0 | L2-7-allT | 6elj_0 | 0 | 1 | 7A |
| 1mam_0 | L2-7-allT | 6j9o_0 | 0 | 1 | 7A |
| 1mcp_0 | L2-7-allT | 1etz_0 | 0 | 1 | 7A |
| 1mlb_0 | L2-7-allT | 5ukn_0 | 0 | 1 | 7A |
| 1nbv_0 | L2-7-allT | 5bvj_3 | 0 | 1 | 7A |
| 1rmf_0 | L2-7-allT | 1etz_0 | 0 | 1 | 7A |
| 1vfa_0 | L2-7-allT | 1a7n_0 | 0 | 1 | 7A |
| 2fb4_0 | L2-7-allT | 5ukn_0 | 0 | 1 | 7A |
| 8fab_0 | L2-7-allT | 7aql_3 | 0 | ? | 7A |
| 2jel_0P (1jel) | L2-7-allT | 5bvj_3 | 0 | 1 | 7A |
| 1yqv_0P (2hfl) | L2-7-allT | 7aql_3 | 0 | 1 | 7A |
| 1bbj_1 | L2-7-allT | 1a7n_0 | 0 | 1 | 7A |
| 2gfb_4 | L2-7-allT | 6j9o_0 | 0 | 1 | 7A |
| 1acy_0P | L2-7-allT | 4g6k_0 | 0 | 1 | 7A |
| 1fbi_1P | L2-7-allT | 1a7n_0 | 0 | 1 | 7A |
| 1fpt_0P | L2-7-allT | 1a7n_0 | 0 | 1 | 7A |
| 1frg_0P | L2-7-allT | 5gs1_2 | 0 | 1 | 7A |
| 1ggi_0P | L2-7-allT | 4g6k_0 | 0 | 1 | 7A |
| 1ikf_0P | L2-7-allT | 1a7n_0 | 0 | 1 | 7A |
| 1jhl_0P | L2-7-allT | 1fve_1 | 0 | 1 | 7A |
| 1ncb_0P | L2-7-allT | 5gs1_7 | 0 | 1 | 7A |
| 3hfm_0P | L2-7-allT | 1a7n_0 | 0 | 1 | 7A |
| 1baf_0H | L2-7-allT | 6q1k_0 | 0 | 1 | 7A |
| 1dbb_0H | L2-7-allT | 1a7n_0 | 0 | ? | 7A |
| 1eap_0H | L2-7-allT | 1a7n_0 | 0 | 1 | 7A |
| 1fig_0H | L2-7-allT | 1a7n_0 | 0 | 1 | 7A |
| 1ibg_0H | L2-7-allT | 6btj_0 | 0 | 1 | 7A |
| 1ind_0H | L2-7-allT | 6j9o_0 | 0 | 1 | 7A |
| 1mfa_0H | L2-7-allT | 5bvj_3 | 0 | 1 | 7A |
| 1tet_0PH | L2-7-allT | 5bvj_3 | 0 | 1 | 7A |
| 2cgr_0H | L2-7-allT | 1a7n_0 | 0 | 1 | 7A |
| 2fbj_0H | L2-7-allT | 3v0v_0 | 0 | 1 | 7A |
| 4fab_0H | L2-7-allT | 1a7n_0 | 0 | 1 | 7A |
| 6fab_0 | L2-7-allT | 2v7h_1 | 1 | 1 | 7B |

No assignments were made for 7fab_0 since it contains an unusual deletion spanning CDR-L2 and framework residues.

Table S3: Clustering results for CDR-L3 loops.

| **AbDb ID** | **LRC** | **AP** | **CAN** | **Chothia** | **M&T1996** |
| --- | --- | --- | --- | --- | --- |
| 1dfb_0 | L3-7-allT | 1dfb_0 | 0 | 4 | 7A |
| 1yqv_0P (2hfl) | L3-8-allT | 3w9d_0 | 0 | 3 | 8A |
| 1eap_0H | L3-8-allT | 3okm_0 | 0 | ? | 8B |
| 1bbd_0 | L3-9-cis95 | 5ezi_0 | 0 | 1 | 9A |
| 1cgs_0 | L3-9-cis95 | 1cgs_0 | 0 | 1 | 9A |
| 1fai_0 | L3-9-cis95 | 3qpq_3 | 0 | 1 | 9A |
| 1for_0 | L3-9-cis95 | 1for_0 | 0 | 1 | 9A |
| 1fvc_0 | L3-9-cis95 | 5vh4_0 | 0 | 1 | 9A |
| 1fvd_0 | L3-9-cis95 | 4ioi_0 | 0 | 1 | 9A |
| 1hil_0 | L3-9-cis95 | 6elj_0 | 0 | 1 | 9A |
| 1igc_0 | L3-9-cis95 | 5x4g_0 | 0 | 1 | 9A |
| 1igf_0 | L3-9-cis95 | 5x4g_0 | 0 | 1 | 9A |
| 1igi_0 | L3-9-cis95 | 6hgu_1 | 0 | 1 | 9A |
| 1igm_0 | L3-9-cis95 | 1jfq_0 | 0 | 1 | 9A |
| 1lmk_0 | L3-9-cis95 | 6hgu_1 | 0 | 1 | 9A |
| 1mam_0 | L3-9-cis95 | 6elj_0 | 0 | 1 | 9A |
| 1mcp_0 | L3-9-cis95 | 1jfq_0 | 0 | 1 | 9A |
| 1mlb_0 | L3-9-cis95 | 1jfq_0 | 0 | 1 | 9A |
| 1nbv_0 | L3-9-cis95 | 5ibu_1 | 0 | 1 | 9A |
| 1rmf_0 | L3-9-cis95 | 6hgu_1 | 0 | 1 | 9A |
| 1vfa_0 | L3-9-cis95 | 1a7n_0 | 0 | 1 | 9A |
| 6fab_0 | L3-9-cis95 | 1jfq_0 | 0 | 1 | 9A |
| 2jel_0P (1jel) | L3-9-cis95 | 4ygv_1 | 0 | 1 | 9A |
| 1bbj_1 | L3-9-cis95 | 1a7n_0 | 0 | 1 | 9A |
| 1acy_0P | L3-9-cis95 | 1a7n_0 | 0 | 1 | 9A |
| 1fbi_1P | L3-9-cis95 | 2fjf_5 | 0 | 1 | 9A |
| 1fpt_0P | L3-9-cis95 | 5ibu_1 | 0 | 1 | 9A |
| 1frg_0P | L3-9-cis95 | 6elj_0 | 0 | 1 | 9A |
| 1ggi_0P | L3-9-cis95 | 6elj_0 | 0 | 1 | 9A |
| 1ikf_0P | L3-9-cis95 | 4ioi_0 | 0 | 1 | 9A |
| 1jhl_0P | L3-9-cis95 | 1a7n_0 | 0 | 1 | 9A |
| 1ncb_0P | L3-9-cis95 | 1a7n_0 | 0 | 1 | 9A |
| 3hfm_0P | L3-9-cis95 | 1ad0_1 | 0 | 1 | 9A |
| 1dbb_0H | L3-9-cis95 | 1ad0_1 | 0 | 1 | 9A |
| 1ibg_0H | L3-9-cis95 | 5ezi_0 | 0 | 1 | 9A |
| 1tet_0PH | L3-9-cis95 | 4ygv_1 | 0 | 1 | 9A |
| 2cgr_0H | L3-9-cis95 | 5ibu_1 | 0 | 1 | 9A |
| 4fab_0H | L3-9-cis95 | 1ad0_1 | 0 | 1 | 9A |
| 1gig_0 | L3-9-allT | 6h3h_1 | 0 | ? | 9D |
| 7fab_0 | L3-9-allT | 3cfj_0 | 0 | 4λ | 9C |
| 1ind_0H | L3-9-allT | 6h3h_1 | 0 | ? | 9D |
| 1mfa_0H | L3-9-allT | 1oaq_0 | 0 | ? | 9C |
| 2gfb_4 | L3-9-allT | 4xbg_1 | 1 | 1 | 9A |
| 8fab_0 | L3-9-allT | 8fab_0 | 3 | ? | 9F |
| 2fbj_0H | L3-9-allT | 3juy_0 | 4 | 2 | 9B |
| 1fig_0H | . | . | . | 1 | 9E |
| 1baf_0H | . | . | . | 5 | 10A |
| 2fb4_0 | L3-11-allT | 6qf9_0 | 2 | 5λ | 11A |

|  | Table S4: Clustering results for CDR-H1 loops. | | | | |
| --- | --- | --- | --- | --- | --- |
| **AbDb ID** | **LRC** | **AP** | **CAN** | **Chothia** | **M&T1996** |
| 1ggi_0P^*^ | H1-12-allT | 5k8a_3 | 3 | 3 | 12A |
| 1bbd_0 | H1-10-allT | 6bi2_0 | 1 | 1 | 10A |
| 1cgs_0 | H1-10-allT | 3cle_0 | 1 | 1 | 10A |
| 1dfb_0 | H1-10-allT | 3cfk_0 | 1 | 1 | 10A |
| 1fai_0 | H1-10-allT | 4qt5_1 | 1 | 1 | 10A |
| 1for_0 | H1-10-allT | 5ob5_0 | 1 | 1 | 10A |
| 1fvc_0 | H1-10-allT | 1fvc_1 | 1 | 1 | 10A |
| 1fvd_0 | H1-10-allT | 6bi2_0 | 1 | 1 | 10A |
| 1gig_0 | H1-10-allT | 6azk_0 | 1 | 1 | 10A |
| 1hil_0 | H1-10-allT | 6pe7_0 | 1 | 1 | 10A |
| 1igc_0 | H1-10-allT | 6ghg_0 | 1 | 1 | 10A |
| 1igf_0 | H1-10-allT | 6pe7_0 | 1 | 1 | 10A |
| 1igm_0 | H1-10-allT | 4z95_0 | 1 | 1 | 10A |
| 1lmk_0 | H1-10-allT | 5wk2_0 | 1 | 1 | 10A |
| 1mam_0 | H1-10-allT | 6nov_0 | 1 | 1 | 10A |
| 1mcp_0 | H1-10-allT | 3pp3_1 | 1 | 1 | 10A |
| 1mlb_0 | H1-10-allT | 6nov_0 | 1 | 1 | 10A |
| 1nbv_0 | H1-10-allT | 1nbv_0 | 1 | 1 | 10C |
| 1rmf_0 | H1-10-allT | 5ob5_0 | 1 | 1 | 10A |
| 1vfa_0 | H1-10-allT | 6azk_0 | 1 | 1 | 10A |
| 2fb4_0 | H1-10-allT | 6ghg_0 | 1 | 1 | 10A |
| 6fab_0 | H1-10-allT | 5wk2_0 | 1 | 1 | 10A |
| 8fab_0 | H1-10-allT | 6pe7_0 | 1 | 1 | 10A |
| 2jel_0P (1jel) | H1-10-allT | 6nov_0 | 1 | 1 | 10A |
| 1yqv_0P (2hfl) | H1-10-allT | 4qt5_1 | 1 | 1 | 10A |
| 1bbj_1 | H1-10-allT | 3pp3_1 | 1 | 1 | 10A |
| 2gfb_4 | H1-10-allT | 4z95_0 | 1 | 1 | 10A |
| 7fab_0 | H1-10-allT | 3pp3_1 | 1 | 1 | 10A |
| 1fbi_1P | H1-10-allT | 1cfq_0 | 1 | ? | 10A |
| 1fpt_0P | H1-10-allT | 3cle_0 | 1 | 1 | 10A |
| 1frg_0P | H1-10-allT | 6ghg_0 | 1 | 1 | 10A |
| 1ikf_0P | H1-10-allT | 4z95_0 | 1 | 1 | 10A |
| 1jhl_0P | H1-10-allT | 3cle_0 | 1 | 1 | 10A |
| 1ncb_0P | H1-10-allT | 3cle_0 | 1 | 1 | 10A |
| 1dbb_0H | H1-10-allT | 5ob5_0 | 1 | 1 | 10A |
| 1eap_0H | H1-10-allT | 6nov_0 | 1 | ? | 10A |
| 1fig_0H | H1-10-allT | 5i16_0 | 1 | 1 | 10D |
| 1ibg_0H | H1-10-allT | 6azk_0 | 1 | 1 | 10A |
| 1ind_0H | H1-10-allT | 1fn4_0 | 1 | ? | 10A |
| 1mfa_0H | H1-10-allT | 5ob5_0 | 1 | 1 | 10A |
| 1tet_0PH | H1-10-allT | 3cle_0 | 1 | 1 | 10A |
| 2cgr_0H | H1-10-allT | 3cle_0 | 1 | 1 | 10A |
| 2fbj_0H | H1-10-allT | 4z95_0 | 1 | 1 | 10A |
| 4fab_0H | H1-10-allT | 5vh4_0 | 1 | 1 | 10A |
| 1igi_0 | H1-10-allT | 1igi_0 | 6 | 1 | 10B |
| 3hfm_0P | . | . | . | 1 | 10A |
| 1baf_0H | H1-11-allT | 5gru_1 | 0 | 2 | 11A |
| 1acy_0P | H1-12-allT | 5k8a_3 | 3 | 3 | 12A |

^*^Note that the Martin and Thornton paper (Table 6) had an error that mislabelled 1ggi as unknown Chothia class and structural cluster 10A instead of class 3 and cluster 12A respectively

|  | Table S5: Clustering results for CDR-H2 loops. | | | | |
| --- | --- | --- | --- | --- | --- |
| **AbDb ID** | **LRC** | **AP** | **CAN** | **Chothia** | **M&T1996** |
| 1gig_0 | H2-9-allT | 1gig_0 | 0 | 1 | 9A |
| 1vfa_0 | H2-9-allT | 1gig_0 | 0 | 1 | 9A |
| 7fab_0 | H2-9-allT | 6z3k_6 | 0 | 1 | 9A |
| 1acy_0P | H2-9-allT | 1fn4_0 | 0 | 1 | 9A |
| 1ggi_0P | H2-9-allT | 1fn4_0 | 0 | 1 | 9A |
| 3hfm_0P | H2-9-allT | 1fn4_0 | 0 | 1 | 9A |
| 1baf_0H | H2-9-allT | 1fn4_0 | 0 | 1 | 9A |
| 1ibg_0H | H2-9-allT | 1fn4_0 | 0 | 1 | 9A |
| 1dfb_0 | H2-10-allT | 6cr1_0 | 0 | 3 | 10B |
| 1hil_0 | H2-10-allT | 6a9k_0 | 0 | 3 | 10B |
| 1igc_0 | H2-10-allT | 6cr1_0 | 0 | 3 | 10B |
| 1igf_0 | H2-10-allT | 6cr1_0 | 0 | 3 | 10B |
| 1igm_0 | H2-10-allT | 6a9k_0 | 0 | 3 | 10B |
| 2fb4_0 | H2-10-allT | 5bvj_3 | 0 | 3 | 10B |
| 8fab_0 | H2-10-allT | 5bvj_3 | 0 | 3 | 10B |
| 2gfb_4 | H2-10-allT | 3cfj_1 | 0 | 3 | 10B |
| 1frg_0P | H2-10-allT | 6hkg_1 | 0 | 3 | 10B |
| 1ikf_0P | H2-10-allT | 6a9k_0 | 0 | 3 | 10B |
| 2fbj_0H | H2-10-allT | 6cr1_0 | 0 | 3 | 10B |
| 1bbd_0 | H2-10-allT | 1ngz_0 | 1 | 2 | 10A |
| 1cgs_0 | H2-10-allT | 2q76_1 | 1 | 2 | 10A |
| 1fai_0 | H2-10-allT | 5i18_0 | 1 | 2 | 10A |
| 1for_0 | H2-10-allT | 6bi0_0 | 1 | 2 | 10A |
| 1fvc_0 | H2-10-allT | 4lly_0 | 1 | 2 | 10A |
| 1fvd_0 | H2-10-allT | 6bi0_0 | 1 | 2 | 10A |
| 1igi_0 | H2-10-allT | 1ngz_0 | 1 | 2 | 10A |
| 1lmk_0 | H2-10-allT | 2q76_1 | 1 | 2 | 10A |
| 1mlb_0 | H2-10-allT | 4lly_0 | 1 | 2 | 10A |
| 2jel_0P (1jel) | H2-10-allT | 4lci_0 | 1 | 2 | 10A |
| 1yqv_0P (2hfl) | H2-10-allT | 2q76_1 | 1 | 2 | 10A |
| 1bbj_1 | H2-10-allT | 1ngz_0 | 1 | ? | 10C |
| 1fbi_1P | H2-10-allT | 1ngz_0 | 1 | 2 | 10A |
| 1fpt_0P | H2-10-allT | 6bi0_0 | 1 | 2 | 10A |
| 1jhl_0P | H2-10-allT | 6al4_0 | 1 | 2 | 10A |
| 1ncb_0P^*^ | H2-10-allT | 4lci_0 | 1 | 2 | 10A |
| 1dbb_0H | H2-10-allT | 5i18_0 | 1 | ? | 10A |
| 1eap_0H | H2-10-allT | 4lly_0 | 1 | 2 | 10A |
| 1fig_0H | H2-10-allT | 2a9m_0 | 1 | 2 | 10F |
| 1mfa_0H | H2-10-allT | 6al4_0 | 1 | 2 | 10A |
| 1tet_0PH | H2-10-allT | 4lci_0 | 1 | 2 | 10A |
| 2cgr_0H | H2-10-allT | 2q76_1 | 1 | 2 | 10A |
| 1ind_0H | H2-10-allT | 4f58_3 | 12 | 3 | 10C |
| 6fab_0 | H2-10-allT | 6fab_0 | 6 | 2 | 10E |
| 1rmf_0 | H2-10-allT | 1rmf_0 | 7 | 2 | 10D |
| 1mam_0 | H2-12-allT | 1mam_0 | 0 | 4 | 12A |
| 1mcp_0 | H2-12-allT | 1mcp_0 | 0 | 4 | 12A |
| 1nbv_0 | H2-12-allT | 7t0k_1 | 0 | 4 | 12B |
| 4fab_0H | H2-12-allT | 3sgd_0 | 0 | 4 | 12B |

^*^Note that the Martin and Thornton paper (Table 6) had an error that mislabelled 1ncb as Chothia class 4 instead of class 2.

# Clustering comparison with North et al. (2011)

Given the time their results were published, there are more structures published. In addition, their study includes bound and unconventional antibodies, which are not included in our study. Therefore, in the following comparison, for each CDR loop, we only compare the clustering results on the intersection of PDB structures found in both studies. This led to a total of 612 CDR loops, ‘H1’: 111, ‘H2’: 102, ‘H3’: 98, ‘L1’: 100, ‘L2’: 101, and ‘L3’: 100.

We compared our canonical clusters, **LRC** and **Canonical** with their **LoopID** and **ClusterID**. In summary, the 612 loops are mapped to 74 of North’s clusters and 103 in ours. Generally, the clustering results are consistent between the two studies. Most of the major clusters are mapped to each other except for some minor clusters that are clustered differently. In Table S6a and S6b, we list the clusters that are a superset of the other study. In general, our clusters are more granular than those from North et al. (2011) , particularly for CDR-H3 loops. This is most likely due to a smaller cutoff of internal distance in our clustering method. The detailed mapping is provided in Supplementary File *Supp08_North_comparison.xlsx*.

The only exception is ‘L1-11-allT’ and Canonical cluster ‘0’ which is a superset of ‘L1-11-1’ and ‘L1-11-2’ in North et al. (2011). This is likely owing to the fact that we employed both internal distance as well as Cartesian distance in our merging criteria, whereas North et al. (2011) only used internal distance. To show that the two clusters do have similar loop shape in Cartesian space, we calculated the pairwise local-fitting C*α* RMSD between the 28 members in ‘L1-11-1’ and 15 ‘L1-11-2’ and found that the average C*α* RMSD is 0*.*38 Å (standard deviation of 0*.*08 Å).

In Table S6a and S6b, for example, a canonical cluster with index ‘1’ in our LRC group ‘L1-11-allT’ is written as ‘L1-11-allT-1’ and a cluster from North et al. (2011) is written as ‘L1-11-1’ (‘LoopID’ of ‘L1-11’ and ‘ClusterID’ of ‘1’). The column ‘PDBid count’ lists the number of ‘PDBid’s find in the corresponding cluster in ‘CL’ or ‘North’.

Table S6: Cluster comparison with North et al. (2011)

| (a)Our clusters that are a superset of clusters from North et al. (2011) | | |
| --- | --- | --- |
| **CL** | **North** | **PDBid_count** |
| H1-10-allT-1 | H1-13-1, H1-13-2, H1-13-4, H1-13-7 | 93, 4, 1, 1 |
| H2-10-allT-0 | H2-10-2, H2-10-4, H2-10-9 | 14, 3, 1 |
| H2-10-allT-1 | H2-10-1, H2-10-3, H2-10-6, H2-10-8 | 53, 4, 1, 1 |
| H3-12-allT-2 | H3-14-1, H3-14-3 | 7, 3 |
| L1-10-allT-0 | L1-10-1, L1-10-2 | 7, 1 |
| L1-11-allT-0 | L1-11-1, L1-11-2 | 28, 15 |
| L2-7-allT-0 | L2-8-1 , L2-8-4 , L2-8-2 , L2-8-3 | 95, 2, 2, 2 |
| L3-8-allT-0 | L3-8-1 , L3-8-2 | 5, 1 |
| L3-9-cis95-0 | L3-9-cis7-1, L3-9-cis7-2, L3-9-cis7-3 | 75, 1, 1 |
|  |  |  |
|  |  |  |
| (b) Clusters from North et al. (2011) that are a superset of our clusters | | |
| **North** | **CL** | **PDBid_count** |
| H1-13-1 | H1-10-allT-1, H1-10-allT-5, H1-10-allT-7 | 97, 1, 1 |
| H1-13-3 | H1-10-allT-0, H1-10-allT-17 | 1, 1 |
| H2-9-1 | H2-9-allT-0, H2-9-allT-1 | 18, 1 |
| H3-10-1 | H3-8-allT-1, H3-8-allT-0 | 4, 4 |
| H3-11-1 | H3-9-allT-9, H3-9-allT-4, H3-9-allT-5 | 2, 1, 1 |
| H3-11-2 | H3-9-allT-10, H3-9-allT-3, H3-9-allT-7, | 1, 1, 1, |
|  | H3-9-allT-0, H3-9-allT-6 | 1, 1 |
| H3-12-1 | H3-10-allT-19, H3-10-allT-4, H3-10-allT-0, | 3, 4, 1, |
|  | H3-10-allT-20, H3-10-allT-11, H3-10-allT-5, | 1, 1, 3, |
|  | H3-10-allT-13, H3-10-allT-15, | 1, 1, |
|  | H3-10-allT-1, H3-10-allT-8 | 1, 1 |
| H3-12-2 | H3-10-allT-7, H3-10-allT-2, H3-10-allT-10 | 2, 1, 1 |
| H3-13-1 | H3-11-allT-14, H3-11-allT-17, H3-11-allT-1 | 1, 1, 1 |
| H3-13-2 | H3-11-allT-13, H3-11-allT-5, H3-11-allT-6, | 2, 1, 1, |
|  | H3-11-allT-3, H3-11-allT-12, H3-11-allT-10, | 1, 1, 1, |
|  | H3-11-allT-8 | 1 |
| H3-14-1 | H3-12-allT-2, H3-12-allT-3, H3-12-allT-0 | 2, 2, 3 |
|  | H3-12-allT-6 | 1 |
| H3-14-3 | H3-12-allT-4, H3-12-allT-2 | 2, 2 |
| H3-16-1 | H3-14-allT-0, H3-14-allT-5, H3-14-allT-7, | 1, 2, 1, |
|  | H3-14-allT-1 | 1 |
| H3-16-2 | H3-14-allT-8, H3-14-allT-9, H3-14-allT-2 | 1, 1, 1 |
| H3-19-1 | H3-17-allT-0, H3-17-allT-1 | 1, 1 |
| H3-24-1 | H3-22-allT-0, H3-22-allT-1 | 1, 1 |
| H3-7-1 | H3-5-allT-0, H3-5-allT-2 | 2, 1 |
| L1-16-1 | L1-16-allT-2, L1-16-allT-7, L1-16-allT-5 | 22, 1, 1 |
| L3-9-2 | L3-9-allT-2, L3-9-allT-1 | 3, 2 |
| L3-9-cis7-1 | L3-9-cis95-0, L3-9-cis95-2 | 76, 1 |

1. Martin, A.C.R. and Thornton, J.M. Structural families in loops of homologous proteins: Automatic classification, modelling and application to antibodies. *Journal of Molecular Biology*, 263(5):800–815, 1996. [↑](#footnote-ref-1)
