# Supplementary material for: Do antibody CDR loops change conformation upon binding?
Source: MAbs. 2024 Mar 13;16(1):2322533. doi: 10.1080/19420862.2024.2322533 (PMC10939163; doi:10.1080/19420862.2024.2322533)
Supplement: Supplemental Material [file KMAB_A_2322533_SM0152.zip › Supp10_PrimaryAuxiliaryComparison--.docx]

Do antibody CDR loops change conformation upon binding?

**Supplementary Material**

Chu’nan Liu, Lilian M. Denzler,
Oliver E.C. Hood, and Andrew C.R. Martin

Structural and Molecular Biology, Division of Biosciences

University College London

Gower Street, London WC1E 6BT

February 7, 2024

**CDR conformational change scale comparison between the auxiliary and the primary dataset**

We added an auxiliary dataset composed of antibodies filtered out during the initial dataset preparation. This includes antibodies that have both VH and VL domains, no CDR missing residues, but at least one CDRα Cα atom with B-factor greater than 80 and resolution worse than 2_._8Å or not specified. This led to 600 AbDb files (details provided in Supplementary File *Supp11_auxiliary_set.xlsx*), which formed 85 groups of unbound and bound antibody pairs. We then performed global and local fitting on this auxiliary dataset following the same procedure as we did for the primary dataset.

# Differences in CDR conformational change on binding

Since the auxiliary dataset is composed of antibodies with poor structure quality, we also mapped the filtered-out antibodies to those in the primary dataset. This would allow us to investigate whether such antibodies in the primary set shows higher CDR conformational change scale than the rest. In total, we found 53 such antibodies and collected their CDR conformational change data from the primary dataset. When compared with the entire primary dataset, we did not find significant difference between the two groups for either global of local fitting (Figure S1a and S1b). *p*-values were calculated using two-sample Mann-Whitney U test and are provided in Table S1.

**(a)**


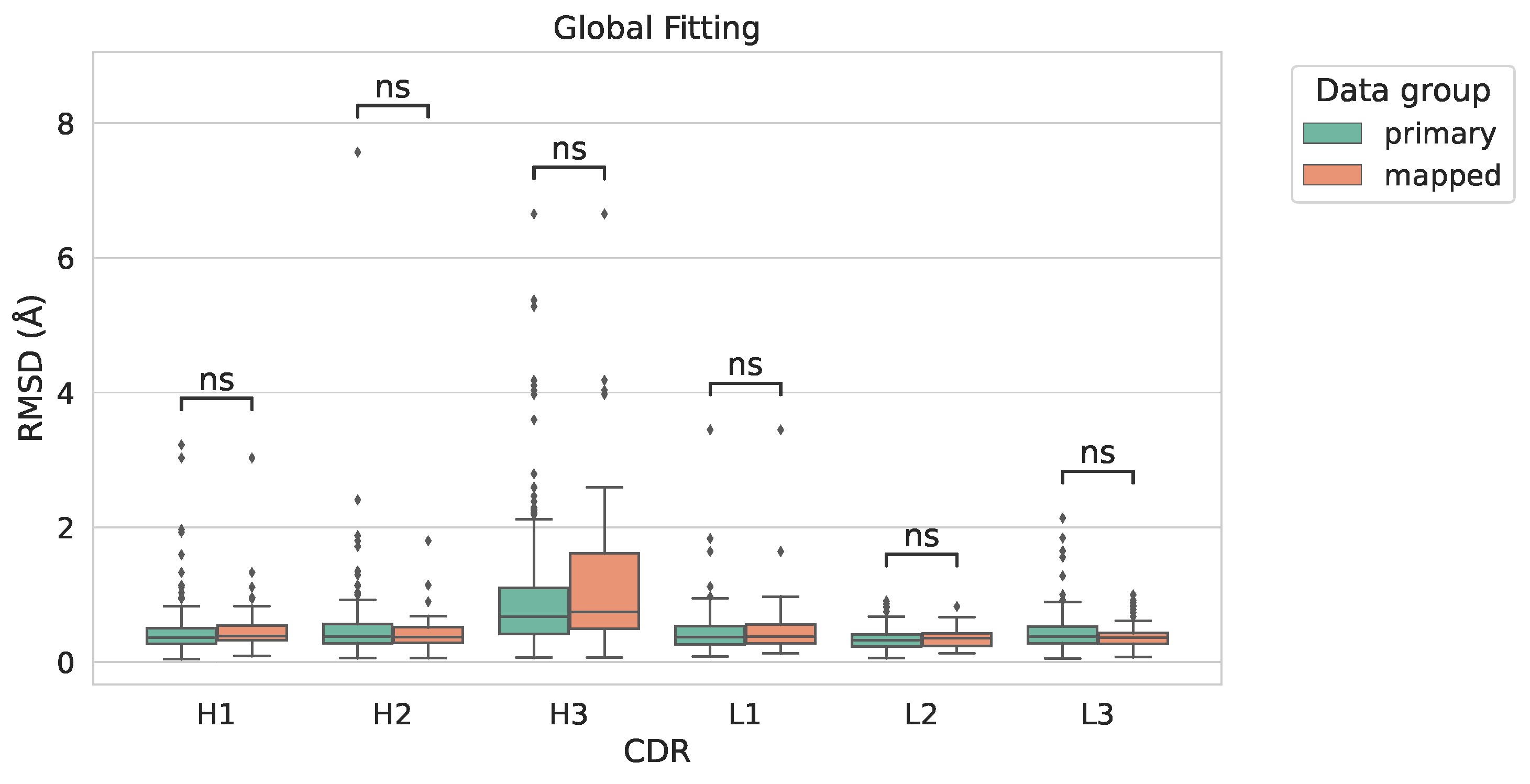


**(b)**


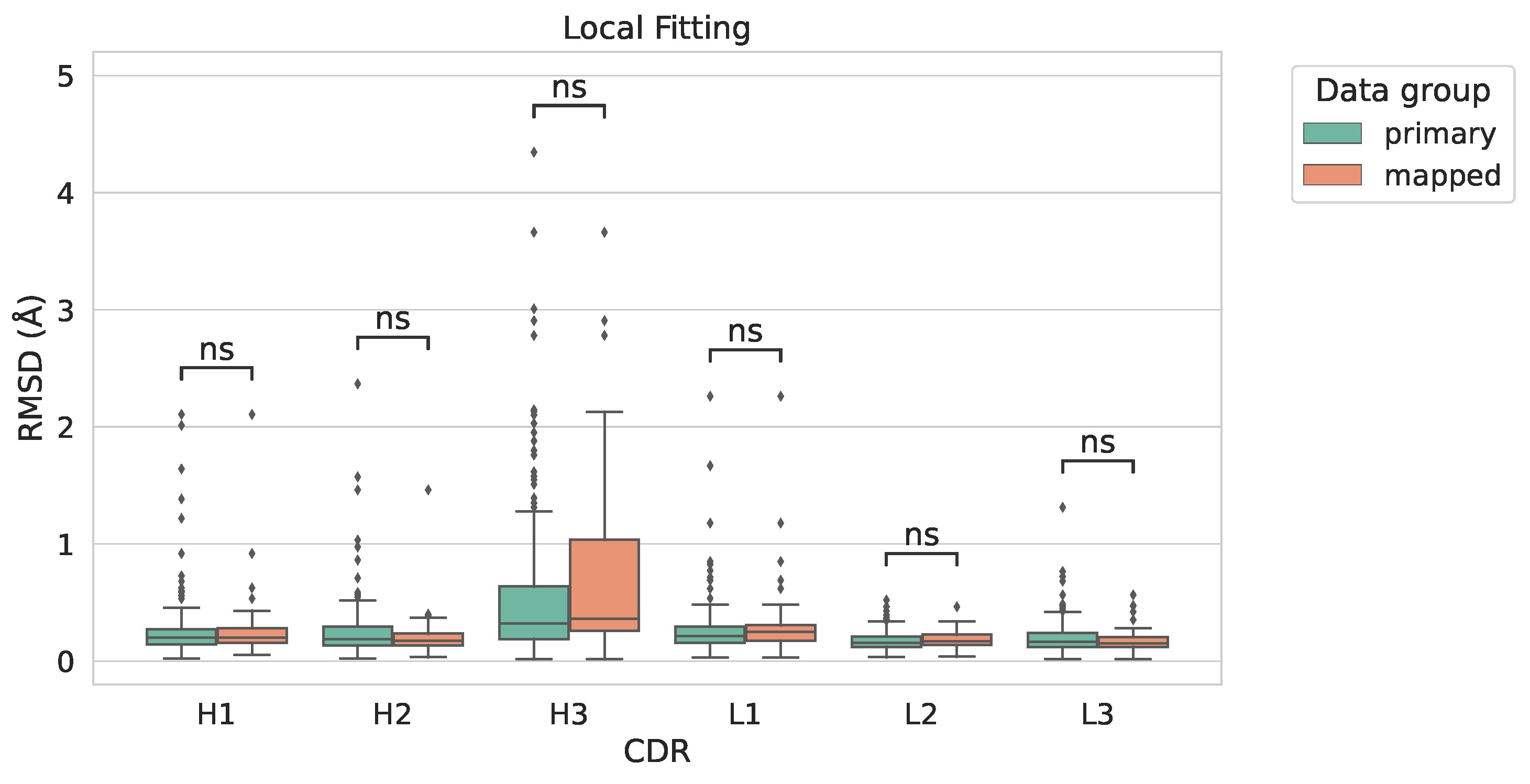


Figure S1: Comparison of CDR conformational change distribution between filtered-out antibodies and the primary dataset. (a) Comparison of CDR conformational change distribution between filtered-out antibodies and the primary dataset from global fitting. The p-value was calculated using two-sample Mann-Whitney U test. ns: not significant. (b) Comparison of CDR conformational change distribution between filtered-outantibodies and the primary dataset from local fitting. The *p*-value was calculated using two-sample Mann-Whitney U test. ns: not significant.

Table S1: CDR conformational change scale comparison

| **CDR** | **Fitting method** | ***p*-value** |
| --- | --- | --- |
| H1 | Global | 0.3801 |
| H2 | Global | 0.5819 |
| H3 | Global | 0.1622 |
| L1 | Global | 0.6270 |
| L2 | Global | 0.4493 |
| L3 | Global | 0.4035 |
| H1 | Local | 0.6262 |
| H2 | Local | 0.3649 |
| H3 | Local | 0.1141 |
| L1 | Local | 0.2038 |
| L2 | Local | 0.2197 |
| L3 | Local | 0.4557 |

# Scale of CDR conformational change

We explored whether the antibodies with high B-factors or low resolution have higher CDR conformational change scales. To this end, we compared the CDR conformational change distribution between the primary dataset and the auxiliary dataset. Generally, the CDR conformational change distribution from the auxiliary dataset is larger than that from the primary dataset for both local and global fitting (Figure S2), except for CDR-H3 loops in global fitting (*p*-value = 0.9838) implying similar CDR conformational change scale of CDR-H3 loops between the two datasets. *p*-values were calculated using a two-sample Mann-Whitney U test and are provided in Table S2.

However, it is worth noting that, the auxiliary dataset is composed of antibodies whose resolution are overall worse than the primary dataset. Over 73% of the 600 AbDb files in the auxiliary set have resolution worse than 2_._8Å. It is hard to determine whether the observed larger CDR conformational change scale is due to the poor structure quality or through binding.

**(a)**


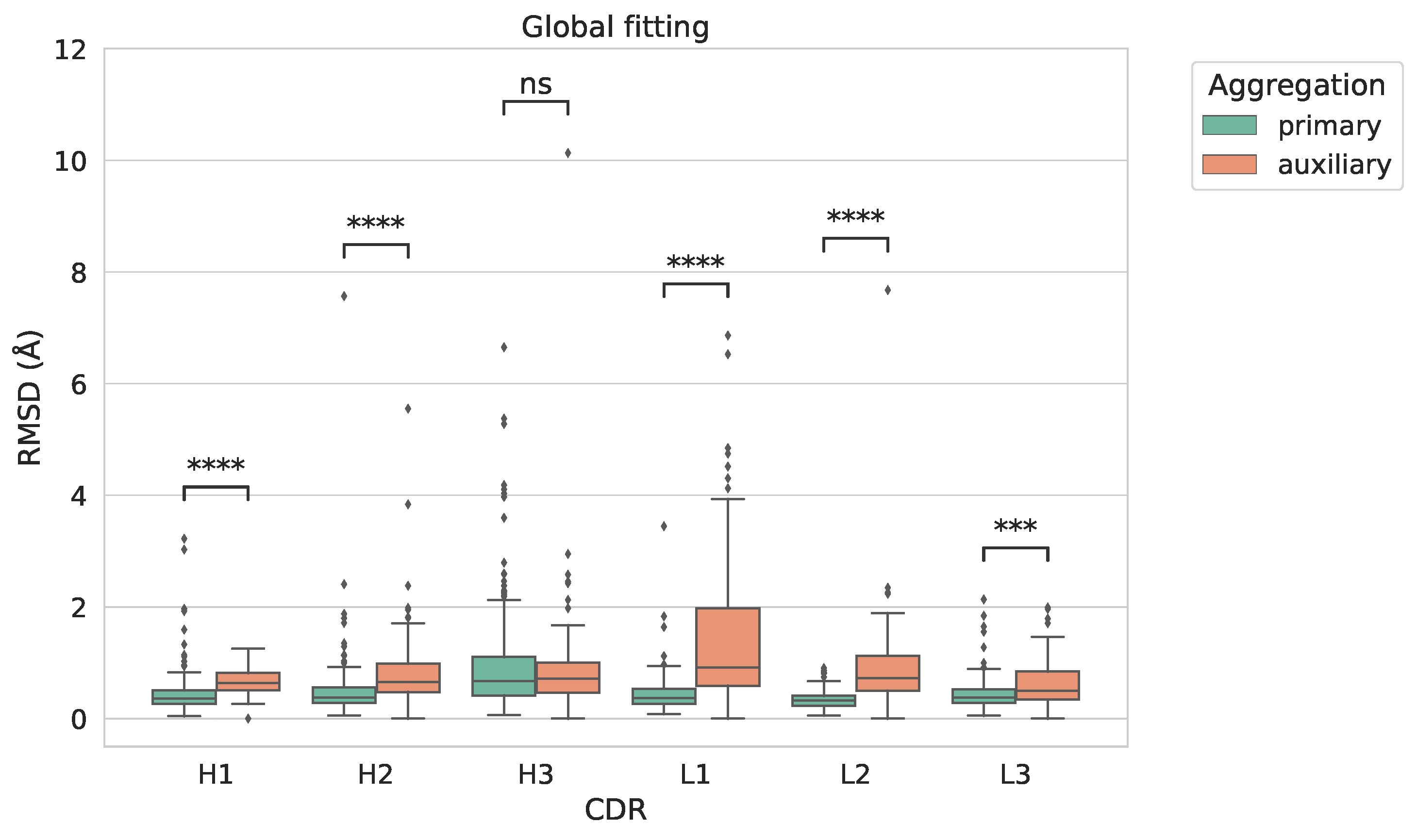


**(b)**


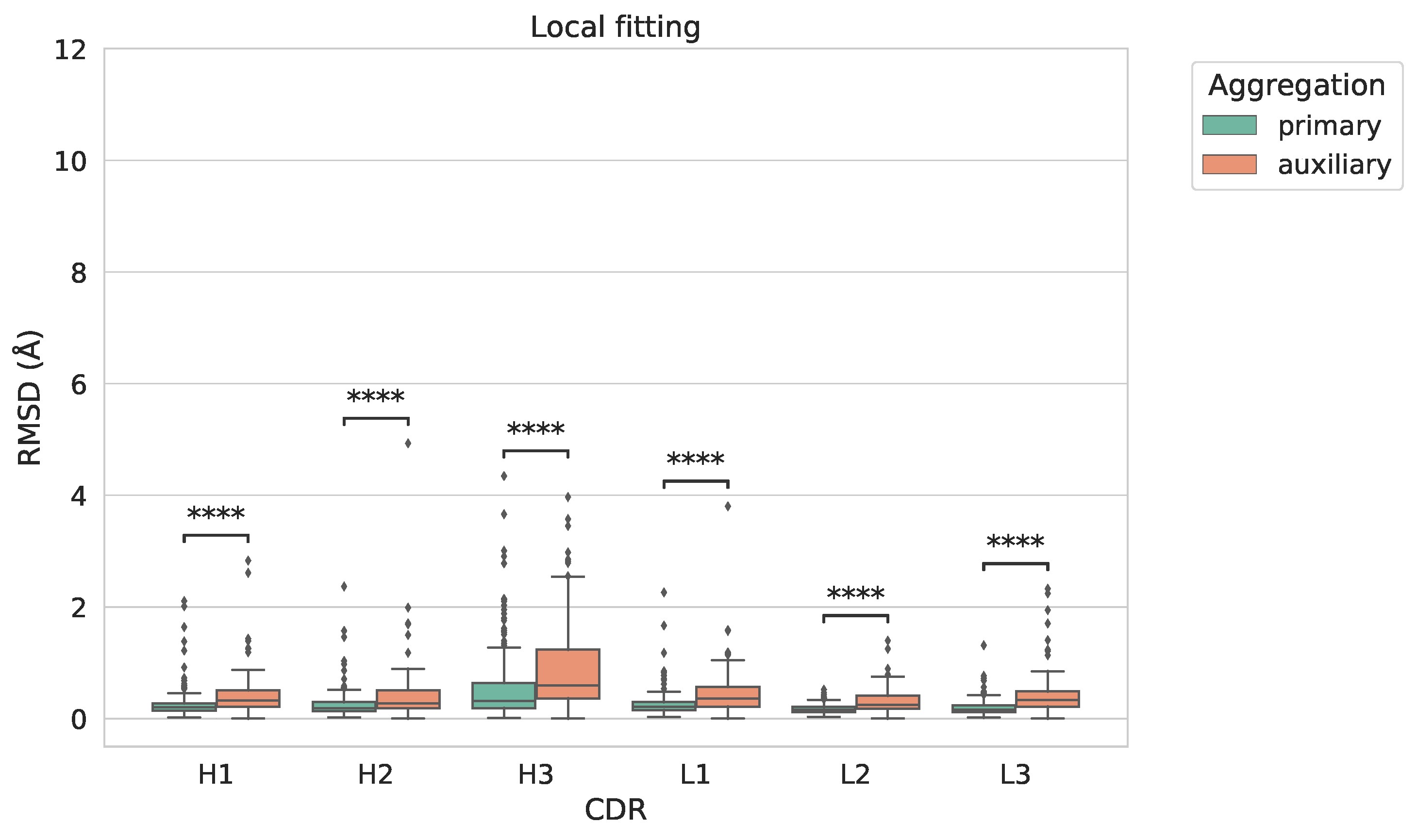


Figure S2: Comparison of primary and auxiliary datasets’ CDR conformational change distributions for local and global fitting. (a) Global fitting CDR conformational change distribution comparison of primaryand auxiliary datasets. *p*-values were calculated using two-sample Mann-Whitney U test. (****: *p*-value ≤ 0.0001, ns: not significant); (b) Local fitting CDR conformational change distribution comparison of primaryand auxiliary datasets. *p*-values were calculated using two-sample Mann-Whitney U test. (****: *p*-value ≤ 0.0001, ns: not significant)

Table S2: CDR conformational change scale comparison

| CDR | Fitting method | *p*-value |
| --- | --- | --- |
| H1 | Global | 6.863 × 10^−12^ |
| H2 | Global | 7.244 × 10^−11^ |
| H3 | Global | 9.838 × 10^−01^ |
| L1 | Global | 9.790 × 10^−20^ |
| L2 | Global | 1.469 × 10^−21^ |
| L3 | Global | 4.395 × 10^−04^ |
| H1 | Local | 2.061 × 10^−06^ |
| H2 | Local | 7.312 × 10^−08^ |
| H3 | Local | 3.647 × 10^−06^ |
| L1 | Local | 5.907 × 10^−09^ |
| L2 | Local | 3.361 × 10^−10^ |
| L3 | Local | 2.047 × 10^−12^ |

Each *p*-value is a comparison between the primary and auxiliary datasets calculated using a two-sample Mann-Whitney U test
